# Non-equilibrium anti-Stokes Raman spectroscopy for investigating Higgs modes in superconductors

**DOI:** 10.1038/s41467-025-62245-4

**Published:** 2025-07-31

**Authors:** Tomke E. Glier, Sida Tian, Mika Rerrer, Lea Westphal, Garret Lüllau, Liwen Feng, Jakob Dolgner, Rafael Haenel, Marta Zonno, Hiroshi Eisaki, Martin Greven, Andrea Damascelli, Stefan Kaiser, Dirk Manske, Michael Rübhausen

**Affiliations:** 1 Institute of Nanostructure and Solid State Physics, Universität Hamburg, Hamburg, Germany; 2 Max Planck Institute for Solid State Research, Stuttgart, Germany; 3 Institute of Solid State and Materials Physics, TUD Dresden University of Technology, Dresden, Germany; 4 Quantum Matter Institute, University of British Columbia, Vancouver, Canada; 5 Department of Physics & Astronomy, University of British Columbia, Vancouver, Canada; 6 Nanoelectronics Research Institute, National Institute of Advanced Industrial Science and Technology, Tsukuba, Ibaraki Japan; 7 School of Physics and Astronomy, University of Minnesota, Minneapolis, USA; 8 Present Address: Heinz Maier-Leibnitz Zentrum (MLZ), Technische Universität München, Garching, Germany; 9 Present Address: Laboratoire MPQ, Université Paris Cité, Paris, France; 10 Present Address: Synchrotron SOLEIL, Saint-Aubin, France

**Keywords:** Superconducting properties and materials, Raman spectroscopy

## Abstract

Even before its role in electroweak symmetry breaking, the Anderson-Higgs mechanism was introduced to explain the Meissner effect in superconductors. Spontaneous symmetry-breaking yields massless phase modes representing the low-energy excitations of the Mexican-Hat potential. Only in superconductors the phase mode is shifted towards higher energies owing to the gauge field of the charged condensate. This results in a low-energy excitation spectrum governed by the Higgs mode. Consequently, the Bardeen-Cooper-Schrieffer-like Meissner effect signifies a macroscopic quantum condensate in which a photon acquires mass, representing a one-to-one analogy to high-energy physics. We report on an innovative spectroscopic technique to study symmetries and energies of the Higgs modes in the high-temperature superconductor Bi_2_Sr_2_CaCu_2_O_8_ after a soft quench of the Mexican-Hat potential. Population inversion induced by an initial laser pulse leads to an additional anti-Stokes Raman-scattering signal, which is consistent with polarization-dependent Higgs modes. Within Ginzburg-Landau theory, the Higgs-mode energy is connected to the Cooper-pair coherence length. Within a Bardeen-Cooper-Schrieffer weak-coupling model we develop a quantitative and coherent description of single-particle and two-particle channels. This opens the avenue for Higgs Spectroscopy in quantum condensates and provides a unique pathway to control and explore Higgs physics.

## Abstract (web summary)

The authors introduce a new spectroscopic technique for studying Higgs modes in superconductors and apply it to a cuprate superconductor. The method involves a soft quench of the Mexican-Hat potential, populating Higgs modes of different symmetries, which are then probed by non equilibrium anti-Stokes Raman scattering.

## Introduction

The Higgs mode is a Raman-active excitation, and was observed in NbSe_2_ in 1980 by R. Sooryakumar and M.V. Klein^1^. The microscopic nature of this discovery was pointed out later by Y. Nambu to P. Higgs, and was seen by both as a first observation of the Higgs mode in experimental physics^2^. However, the Raman cross section of the Higgs mode in superconductors is generally very small, which results in Higgs modes that remain invisible in most Raman experiments. In NbSe_2_, a unique interplay between a soft phonon in the charge-density wave state and the Higgs mode in the superconducting (SC) state leads to a distinct and sharp mode slightly below 2Δ^3–5^. This interplay has been explored as a function of temperature and pressure^6,7^, and more recently, the coupling between charge-density wave and Higgs mode has also been studied by time-resolved spectroscopy of amplitude- and phase-sensitive high harmonics^3,8^. Up to now, the observation of the Higgs mode in Raman scattering has been limited to the particular case of NbSe_2_. In non-charge-density-wave systems, the Mexican-Hat potential must be specifically quenched, for instance by a light pulse, to make the Higgs mode observable^9^.

Spontaneous Raman spectroscopy has been widely used to study excitation-energy resonances and the dynamics of the SC gap feature in high-temperature superconductors. Static Raman spectra of Bi_2_Sr_2_CaCu_2_O_8+*δ*_ (Bi-2212) in *B*_1g_ symmetry show distinct resonances between 2 eV and 3.5 eV, indicating a multicomponent origin of the excitation spectrum close to 2Δ^10^. Furthermore, transient time-resolved Raman scattering in the SC state enables the study of pair-breaking (PB) excitations and the dynamics of the SC order parameter^11^. By utilizing the Bose factor, time-resolved Stokes-anti-Stokes Raman scattering has been applied as a stroboscopic tool to determine transient temperatures and melting processes in highly excited states of phonons^12,13^.

Advances in THz-laser technology have enabled the investigation of Higgs modes in several classes of superconductors via non-equilibrium THz spectroscopy^9,14–21^. These experiments involve either an impulsive excitation of the Higgs mode based on quench of the SC state or a drive of the Higgs mode, resulting in coherent oscillations or high-harmonic generation, respectively. Experiments on s-wave superconductors confirm that Higgs modes are stable excitations^20^, whereas in d-wave superconductors they are metastable due to interactions with remanent nodal quasiparticles^19,22,23^, resulting in a spectral broadening of the Higgs mode. The theoretical models are based on time-dependent Ginzburg-Landau approaches and time-dependent BCS theories in the framework of a pseudospin model^16,23–25^. Additionally, the important influence of disorder and impurity effects on high-harmonic generation was investigated from a theoretical point of view^26–28^. Furthermore, the SC state exhibits a manifold of low-energy excitations, such as pair breaking, Josephson plasmons, Bardasis-Schrieffer, or Leggett modes^22,29–31^. Some of them are difficult to distinguish from the Higgs mode in an experimental data set. Thus, it is of great importance that the Higgs modes can be classified based on the symmetry of the SC condensate, the symmetry of the quench, and the symmetry of the Higgs excitation^32^. This unique diversity of physical properties enables the exploration and control of Higgs physics in an unprecedented way and inspires the development of a transient symmetry-sensitive spectroscopic technique.

Here, we introduce a new spectroscopic technique: Non-Equilibrium Anti-Stokes Raman Scattering (NEARS). NEARS utilizes a so-called soft quench of the Mexican-Hat potential, as we will detail below, with the goal of populating Higgs modes of different symmetries, which are then probed by anti-Stokes Raman scattering. The conventional spontaneous Raman scattering signal is proportional to a four-photon Green’s function where Stokes and anti-Stokes signals are generated simultaneously. Compared to conventional Raman scattering, non-equilibrium Raman scattering overpopulates the lowest-energy excitation of the superconductor, which is the metastable Higgs mode, as a consequence of the relaxation process of the free-energy landscape. This population inversion (see SI Fig. S9) can be measured by a comparison between the simultaneous Stokes and anti-Stokes signals, i.e., energy-loss and energy-gain data of the spontaneous Raman process.

## Results

**Fig. 1:**
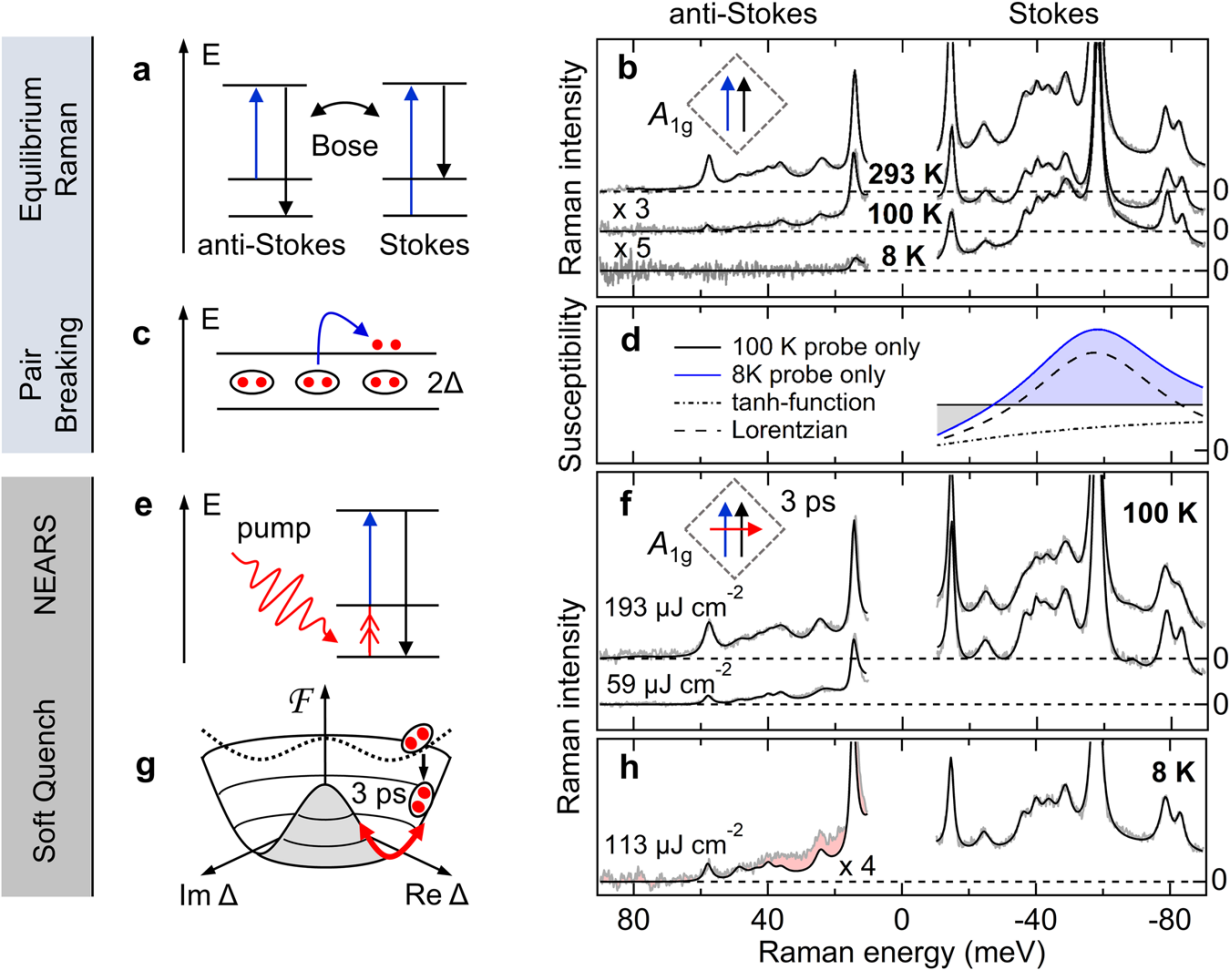
Equilibrium vs. non-equilibrium resonant Raman scattering. **a** Energy diagrams of Stokes and anti-Stokes Raman scattering. The scattering cross-sections of equilibrium Stokes (energy-loss) and anti-Stokes (energy-gain) scattering are linked by the Bose function. The Raman-probe excitation (blue) and scattered light (black) are depicted. Please note that this schematic is not drawn to scale. In all measurements, the Raman-probe excitation is 3 eV, while the Raman shift is in the meV range. **b** Equilibrium Raman data of Bi-2212 (*T*_c_ = 92 K^38^, see Fig. S3) in an *A*_1g_ scattering configuration at the indicated base temperatures. Measured intensities (gray) are fitted (black solid lines, see SI S.6). The dashed lines represent zero intensity for the three displayed data sets. The anti-Stokes Raman spectrum at 8 K is vanishing because the anti-Stokes Bose-function at 8 K is infinitesimally small. **c** The Raman response is dominated by PB of Cooper pairs. **d** Electronic Raman susceptibility in probe-only measurements as extracted from the parameterization (see SI S.6, Fig. S10). At 100 K the electronic background is constant (black solid line) within the presented energy range. The applied tanh function with a small *ω*_*C*_ approaches zero at Raman shifts below 10 meV. At 8 K, the PB feature dominates the response (blue line). The PB feature consists by a tanh-function (dash-dotted line) and a Lorentzian (dashed line). **e** After a pump (red wiggly line), the populated Higgs mode (red double arrow) can be detected by an annihilation process (anti-Stokes). **f** Pump-probe Stokes and anti-Stokes Raman spectra at a fluence of 59 μJ cm^−2^ and 193 μJ cm^−2^ (3ps delay, *A*_1g_, 100 K base temperature). **g** The pump-induced modification of the free-energy landscape (dotted line) enables annihilation of Higgs oscillations (solid red line) at 3 ps delay. **h** Below T_c_ (8 K base temperature), at an exemplary fluence of 113 μJ cm^−2^ the anti-Stokes intensity cannot be described via the Stokes susceptibility and a new NEARS feature occurs (red), which we attribute to the *A*_1g_ Higgs mode. Please note that at low temperatures, higher fluences were not applied to remain in the SC state.

Conventional Raman scattering excites quasiparticles leading to energy-loss spectroscopic features on the Stokes side (see Fig. 1a–d). In superconductors, this technique is sensitive to low-energy excitations, such as PB excitations^33,34^, see Fig. 1c, d, density-correlation functions of Josephson plasmons^31^, Leggett modes^35^, and Bardasis-Schrieffer modes^36,37^. Our aim is to measure the relaxation of a superconductor and the concomitant population of Higgs modes in the quasi-static limit and in nearly thermal equilibrium. As a result, excitations that are not the lowest-energy metastable states will decay more rapidly than the Higgs mode and thus remain undetectable in NEARS. This strongly supports the Higgs mode’s relevance in interpreting any additional features observed in NEARS measurements.

In this work, NEARS is used to study the high-temperature superconductor Bi-2212 (optimally doped, T_c_ = 92 K, see SI Fig. S3)^38^. To verify the condition of quasi-equilibrium in the non-pumped data, we use the fact that Stokes and anti-Stokes scattering intensities in equilibrium are linked by the Bose function. We measure anti-Stokes and Stokes data and convert the Stokes spectrum to the corresponding anti-Stokes response by applying the Bose function. Matching Stokes and anti-Stokes spectra over a large low-energy range ensures that we have determined the temperature of the sample under laser illumination, excluded any background artifacts from the pulsed laser source, and avoided unwanted self-excitation effects induced by the pulsed Raman probe (see Figs. S5 and S8)^39^. Indeed, in Fig. 1b, a perfect agreement between the converted and measured anti-Stokes data is observed when assuming a heating of 9 K due to the Raman probe. We apply a scattering geometry that probes *A*_1g_ symmetry with incidence and scattered light fields parallel to each other and rotated 45° to the CuO_2_ plane of Bi-2212, as indicated in the pictogram of Fig. 1b. An incident photon energy of 3 eV (400 nm), a laser power of 4.8 mW, and a pulse duration of 1.2 ps were used. In the SC state, at low temperatures, and on the Stokes side, we observe the well-known phonons and excitations around twice the SC gap energy (2Δ ≈ 60 meV) due to the predominant PB process of Bi-2212 as expected for a sample with T_c_ ≈ 92 K^10,40^. As shown in Fig. 1d, the Raman susceptibility of the PB feature is described by a tanh-function and a Lorentzian according to^10^. The PB process is sketched in Fig. 1c. At 8 K, the anti-Stokes side is essentially dark as no quasiparticles are thermally excited at T  ≈ 0 K and, hence, no annihilation of excitations can occur (see 8 K data in Fig. 1b).

The NEARS experiment is illustrated in Fig. 1e. The pump quenches the Mexican-Hat potential, but allows it to relax so that the Higgs mode populates as shown in Fig. 1g. We call this scenario a soft quench, which controls the inversion population of the metastable Higgs mode via the fluence. In the normal state (Fig. 1f), with a pump (1.2 ps, 1.55 eV (800 nm)) orthogonal to the Raman probe polarizations and with a time delay of 3 ps between pump and probe, we can still apply the quasi-equilibrium approach. We can convert the measured Stokes spectra to the measured anti-Stokes data by using an additional pump heating of 3 K/mW (see Fig. S8). This is in contrast to many other experiments that use peak powers three to five orders of magnitude higher and thus explore the physics of hot electrons and hot phonons in the hard quench regime^41–44^. At 3 ps delay, fast electronic and phononic responses have already decayed^41,43,44^. Thus, following Kasha’s Rule, the soft quench populates the lowest metastable excitation of the superconductor, which is the Higgs mode^45^.

Indeed, in the SC state we identify an extra signal on the anti-Stokes side (see Fig. 1h) which can be assigned to the population of the Higgs modes. This feature is solely present on the anti-Stokes side, while on the pumped Stokes side we can identify a persistent suppressed PB peak, clearly indicating that the sample is still in its SC state 3 ps after the pump (see also S5 and Fig. S12). We argue in the SI (S.7) that alternative excitations such as Josephson plasmons and Bardasis-Schrieffer modes cannot be responsible for our observations. Instead, the experimental results are in agreement with Higgs excitations. Within the phenomenological Ginzburg-Landau theory, we can connect the energy of the Higgs excitation to the Cooper-pair coherence length. Within a BCS weak-coupling model, we further develop a quantitative and coherent description of the pair-breaking excitations in the single-particle channel together with the Higgs excitations in the two-particle channel.

**Fig. 2:**
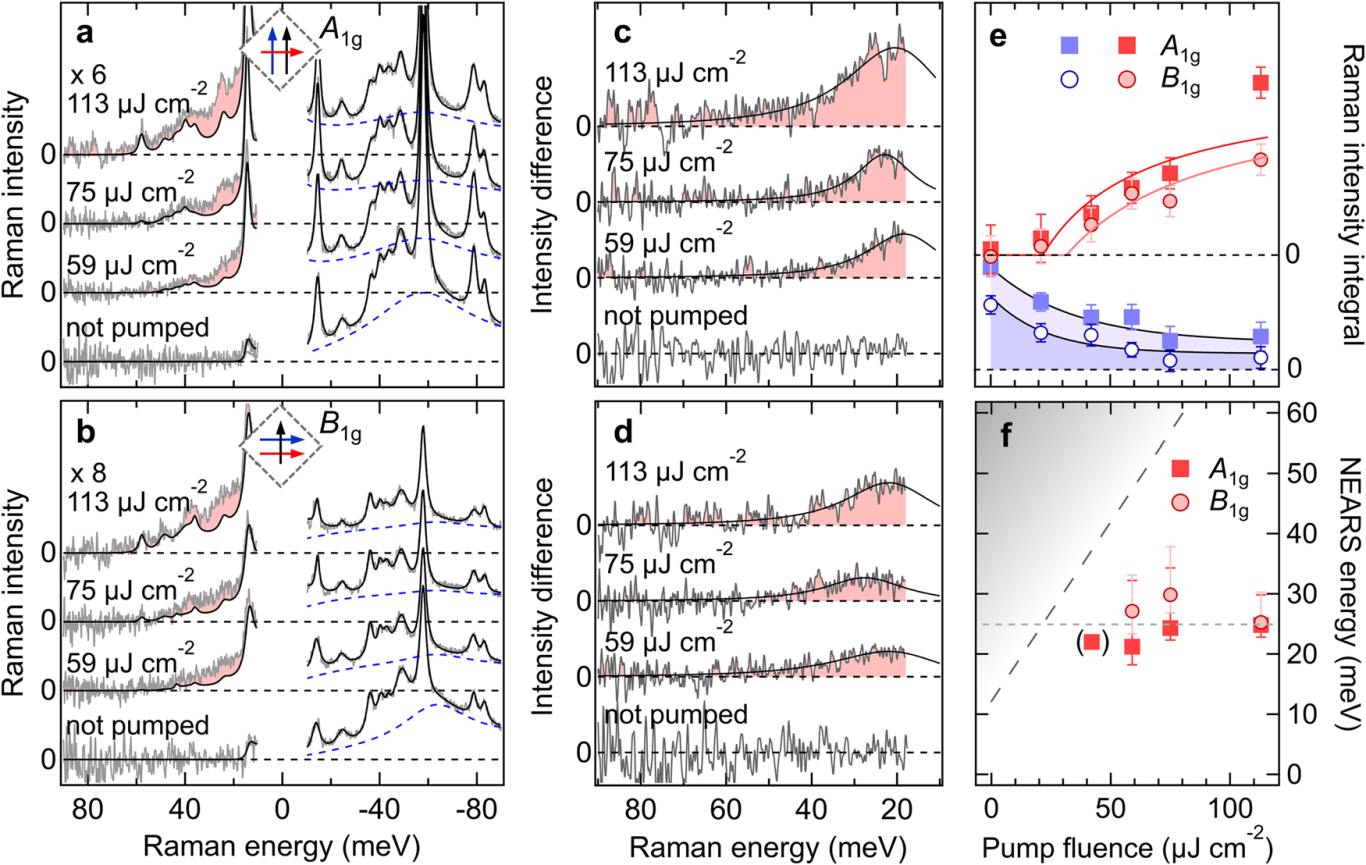
Higgs modes as a function of fluence. **a** *A*_1g_ Pump-probe Stokes and anti-Stokes Raman spectra at 8 K base temperature and a time delay of 3 ps for selected fluences between 0 μJ cm^−2^ (not pumped) and 113 μJ cm^−2^ (see Fig. S6 for complete data set). Anti-Stokes intensities are scaled by a factor of 6 for visibility. Data (gray) and fits (black, see SI S.6) are shown, with dashed lines marking zero intensity. The parameterized PB response on the Stokes side is highlighted in blue. With increasing fluence, gap filling occurs, and PB amplitude decreases. Anti-Stokes spectra are shown with Stokes-side fits converted via the Bose function (black lines). Quasi-equilibrium temperatures used for the fits are listed in Table S2. At fluences larger than 50 μJ cm^−2^ the in-gap electronic response is dominated by a new NEARS feature (*A*_1g_ Higgs mode), resulting in a difference signal compared to the Stokes signal (red). **b** *B*_1g_ Pump-probe Stokes and anti-Stokes Raman spectra at 8 K analogous to (**a**). **c** *A*_1g_ difference signal between anti-Stokes data and Stokes-fit (corresponding to red area in a). Solid black lines represent fits following eq. (1) (see text and SI S.4). **d** *B*_1g_ difference intensities analogous to (**c**). **e** Integrated Raman intensities of the Higgs modes (red) and of the PB response (blue) as a function of fluence. The Higgs mode intensity integrals are the integrated anti-Stokes difference spectra shown in (**c**) and (**d**). Error bars are determined based on the noise of the integrated data. Solid black lines are exponential guides to the eye. The red lines represents the equation of inversion population (see SI S.3) with a critical fluence of 21.7 ± 5.3 μJ cm^−2^ for *A*_1g_ and 31.6 ± 2.3 μJ cm^−2^ for *B*_1g_ symmetry. **f** Excitation energy of the Higgs modes vs. fluence. Error bars show the standard uncertainty of the fitted parameter ${\omega }_{H}=\sqrt{2\alpha }$ (see eq. 1). The equilibrium anti-Stokes intensity is limited at higher energies due to thermal Bose factors of the Raman intensity (gray-shaded area, see also Fig. S4) leading to asymmetric error bars.

Figure 2 presents the fluence dependence of the Bi-2212 NEARS spectra at 8 K and at 3 ps delay in *A*_1g_ and *B*_1g_ symmetry, as shown in the pictograms of Fig. 2a, b. The dashed squares mark the orientation of the CuO_2_ planes. The pump is applied along the diagonals of the CuO_2_ planes and carries a non-zero in-plane momentum due to its incidence angle of 21.8° (see Fig. S2)^46^. In this configuration, one expects Higgs modes in both *B*_1g_ and *A*_1g_ probe symmetries^32^. The direct comparison between Stokes and anti-Stokes responses allows us to discriminate excitations around 2Δ from NEARS features below 2Δ. Figure 2a, b show Stokes and anti-Stokes Raman intensities for exemplary fluences between 0 μJ cm^−2^ (not pumped) and 113 μJ cm^−2^. Raman intensities corresponding to the parameterized PB feature on the Stokes side are depicted in blue. One can clearly see that the excitations around 2Δ in both *B*_1g_ and *A*_1g_ Raman probe symmetry get suppressed with increasing fluence, but remain non-zero even at the highest fluence, which demonstrates that Bi-2212 remains in the SC state (see also SI S.5 and Fig. S12). The anti-Stokes spectra are presented together with the phononic and electronic Raman intensity fitted to the Stokes side and converted to the anti-Stokes side by the Bose function. The utilized quasi-equilibrium temperatures are listed in Table S2 (see also SI S.5) and are confirmed by evaluating the superconductivity-induced features of the Stokes spectrum as a function of fluence (see Fig. S12). The additional signal, which we attribute to the Higgs mode is highlighted in red. This feature on the anti-Stokes side increases in intensity with increasing fluence. We associate this with the enhanced inversion population of the metastable Higgs mode as a consequence of the increased strength of the soft quench. In a three-level picture of population inversion (shown in Fig. S9a), an excited state is populated with a short lifetime *τ*_relax_^41^ by quenching the Mexican-Hat. Subsequently, a metastable lower-energy state (i.e., the Higgs mode) with a longer lifetime *τ*_Higgs_ > *τ*_relax_ is populated. Population inversion occurs if *N*_Higgs_ > *N*_initial_. After exceeding a critical fluence that is required for population inversion, the anti-Stokes intensity of the metastable Higgs excitation scales with the ratio (*N*_Higgs_ − *N*_initial_)/*N*_total_.

Figure 2c,d show the differences between the anti-Stokes data (gray in a and b) and converted Stokes fits (black solid lines in a and b), respectively. In Fig. 2e, we show the integrated Raman intensity of the Higgs modes and the PB excitation as a function of fluence. In both probe symmetries, we find an increase of the Higgs mode intensity and a concomitant decrease of the PB. The integrated intensities of the anti-Stokes Higgs difference signals as a function of fluence agree very well with the equation of population inversion (red lines) with a critical fluence of 21.7 ± 5.3 μJ cm^−2^ for *A*_1g_ and 31.6 ± 2.3 μJ cm^−2^ for *B*_1g_ symmetry (see SI S.3, eq. S24, and Fig. S9b).

## Discussion

A phenomenological model of the Higgs response can be obtained within Ginzburg-Landau theory utilizing a Klein-Gordon like Lagrangian. The equations of motion are derived from a Mexican-Hat potential $F(\Psi )=\alpha | \Psi {| }^{2}+\frac{\beta }{2}| \Psi {| }^{4}$ (*α* < 0) with amplitude and phase fluctuations^47^. Since the phase fluctuations are gauged out by the Anderson-Higgs mechanism, we can calculate the Green’s function of the Higgs mode through the equations of motion in the optical **q** → 0 limit assuming a *δ* function-like quench due to a change in *β* (see SI S.4). The result is a Lorentzian response *I*(*ω*)

1 $$I(\omega )={I}_{0}\frac{\gamma \omega }{{({\omega }^{2}-2| \alpha | )}^{2}+{(\gamma \omega )}^{2}}\,,$$

where *γ* is the phenomenological width and 2*α* corresponds to the energy of the Higgs mode. The SC coherence length $\xi=\sqrt{{\hslash }^{2}/(| \alpha | 4{m}^{*})}$ is given by the Higgs-mode energy *α*.

Figure 2f shows the fluence dependence of the symmetry-dependent excitation energies derived by fitting the NEARS difference data with eq. (1) as shown in Fig. 2c, d. In *A*_1g_ and *B*_1g_ symmetry the Higgs mode arises at around 2*α* = 0.168 ⋅ 2Δ_0_ = 10.24 meV, corresponding to an energy of ${\omega }_{H}=\sqrt{2\alpha }=0.41\cdot 2{\Delta }_{0}=25$ meV. Using established values m*/*m*_*e*_ for optimally-doped cuprates of the order of m* = 10*m*_*e*_^48^, we obtain in-plane coherence lengths of smaller than 5 nm in agreement with other experimental observations^49^. The Higgs-mode energy is only weakly dependent on fluence as expected for a population quench.

**Fig. 3:**
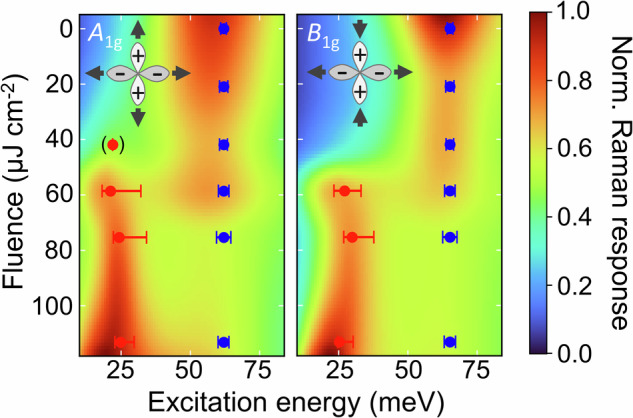
Unified excitation spectrum of the superconductor Bi-2212. The NEARS maps of Bi-2212 show the Raman response of the Higgs mode at around 25 meV together with the PB excitation Raman susceptibility around 60 meV in *A*_1g_ (left) and *B*_1g_ (right) geometry at a time delay of 3 ps. To create the NEARS maps, the fits from Fig. 2 were interpolated as a function of fluence. Raman energies of the utilized Lorentzians are shown as red and blue data points, respectively. Error bars are the standard uncertainty of the fitted frequency for the Higgs mode and PB peak, respectively. The symmetry of the Higgs modes for the respective pump/probe configuration is indicated at the top left.

In Fig. 3, we jointly show the PB excitations of the single-particle channel together with the Higgs modes of the two-particle channel (see also Figs. 1, 2 and Fig. S7) yielding a unified excitation landscape of the SC state in Bi-2212. These NEARS maps represent a superposition of the fluence-dependent superconductivity-induced excitations from both the Stokes and anti-Stokes spectra in *A*_1g_ and *B*_1g_ symmetry, respectively, identifying the *A*_1g_ and *B*_1g_ Higgs modes. The non-quenched energy landscape is dominated by excitations around 2Δ^31,33^. With increasing fluence, the 2Δ excitations gradually weaken, but remain at constant energy, indicating that Bi-2212 remains in its SC state. The Higgs mode is an in-gap excitation around 0.4 2Δ and increases in strength with fluence, showing a broadening at the highest fluence.

To interpret the NEARS data in a microscopic framework, we utilize a mean-field weak-coupling BCS theory, as discussed in detail in SI S.2. The superconductor exhibits two main contributions to its electronic Raman response in the energy range of the gap: the quasiparticle/PB response due to single-particle excitations and the collective modes’ response^50^. The Higgs mode is the lowest-energy excitation in the two-particle channel and therefore plays a prominent role. The PB Raman vertices for *B*_1g_ and *A*_1g_ can be expressed as ${\gamma }_{{B}_{1{{\rm{g}}}}}={\gamma }_{b}\cos (2\phi )$ and ${\gamma }_{{A}_{1{{\rm{g}}}}}=(1+{b}_{0}\xi )({\gamma }_{0}+{\gamma }_{1}\cos (4\phi )+{\gamma }_{2}\cos (8\phi ))$, respectively^34^. *γ*_0_, *γ*_1_, *γ*_2_ are the coefficients in the generalized Fermi-surface harmonic expansion of ${\gamma }_{{A}_{1{{\rm{g}}}}}$, and *γ*_*b*_ is the coefficient in the expansion of ${\gamma }_{{B}_{1{{\rm{g}}}}}$. We choose polar coordinates whose angle *ϕ* is defined as described in Fig. S14. The energy dependence of ${\gamma }_{{A}_{1{{\rm{g}}}}}$ is encoded in *b*_0_. The deviation from a constant density of state in the thin energy hull around the Fermi surface, inside which we assume the net-attractive electron-electron interaction, is represented by *b*_1_: *N*(*ξ*) = *N*_*F*_(1 + *b*_1_*ξ*). Please note that *b*_0_ and *b*_1_ break particle-hole symmetry in the Raman vertices and the density of states, accordingly. Within BCS theory, the breaking of particle-hole symmetry is required in order to obtain a finite cross-section of the Higgs mode.

**Fig. 4:**
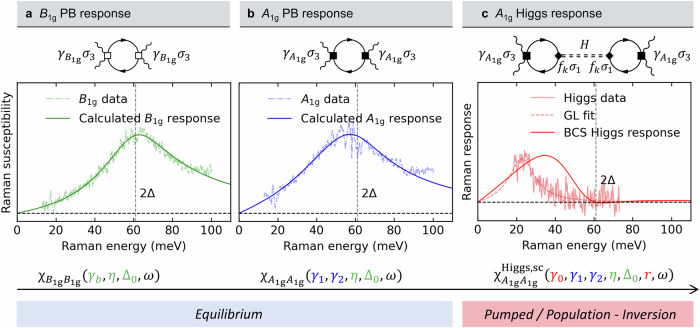
Comparison between NEARS data and BCS model calculations. **a** The experimental *B*_1g_ electronic Raman susceptibility on the Stokes side (dash-dotted line) is extracted by parameterization and phonon subtraction as described in SI S.6. The *B*_1g_ PB Raman response is depicted diagrammatically in the upper part of panel **a** (see also eq. S13). We find the parameters *γ*_*b*_ = 0.0789 ± 0.0002, *η* = 0.225 ± 0.003, and Δ_0_ = 30.50 ± 0.05 meV from the *B*_1g_ fit (green). **b** The *A*_1g_ PB Raman response is depicted diagrammatically with filled squares representing the Coulomb-screened vertices (see also eq. S17). We keep *η* and Δ_0_ from the *B*_1g_ fit, and fit *γ*_1_ and *γ*_2_ to the *A*_1g_ experimental data (blue, *γ*_1_ = 0.0825 ± 0.0007, *γ*_2_ = 0.0385 ± 0.0006 with *η* = 0.225, Δ_0_ = 30.50 meV.). Please note that no information of *γ*_0_ can be obtained due to the complete screening of the isotropic *A*_1g_ component^34^. We assume that *b*_0_ and *b*_1_ are small (Δ_0_*b*_0_, Δ_0_*b*_1_ ≪ 1), therefore *A*_1g_ has negligible dependence on *b*_0_, *b*_1_. **c** Finally, the *A*_1g_ Higgs response is evaluated with the free parameters *γ*_0_ and *r* = *b*_1_/(*b*_0_ + *b*_1_) (see Feynman diagram and eq. S21). All other parameters (*γ*_1_, *γ*_2_, *η*, Δ_0_) are fixed. The red solid line corresponds to *γ*_0_ = 0.071 and *r* = 0.88. The experimental Higgs Raman response corresponds to the 75 μJ cm^−2^ *A*_1g_ data presented in Fig. 2c. The dashed line represents the fit according to eq. (1) derived within Ginzburg-Landau theory. Please note that due to the population of the Higgs mode by the soft quench, the Higgs response is a non-equilibrium response and enhanced in our experiment compared to the model. We therefore scale the intensities accordingly.

In Fig. 4a, b we compare the results from our calculations with the equilibrium experimental data. In a first step, the lowest-order contribution to the *B*_1g_ PB susceptibility is evaluated as shown in the Feynman diagram of Fig. 4a (see SI eq. S13)^34^. We obtain an excellent agreement between the parameterized, phonon-subtracted quasiparticle susceptibilities from unpumped measurements and the microscopically calculated quasiparticle response for a coefficient *γ*_*b*_ = 0.0789 ± 0.0002, an electronic lifetime *η* = 0.225 ± 0.003 corresponding to 2Δ_0_*η* = 13.5 meV, and a superconducting order parameter Δ_0_ = 30.50 ± 0.05 meV. Since *η* and Δ_0_ are expected to be shared amongst all response functions, we keep *η* and Δ_0_ constant for all further calculations.

NEARS utilizes a probe energy of 3.1 eV. It is known that the PB peak changes as a function of incident photon energy^10^. We model the Coulomb-screened *A*_1g_ response in terms of Fermi-surface harmonics with the expansion parameters *γ*_1_ and *γ*_2_ (see SI S.2.4). Diagrammatically, the *A*_1g_ PB response modified by Coulomb screening is shown in Fig. 4b (see eq. S17). Fitting *γ*_1_ and *γ*_2_ to the experimental phonon-subtracted *A*_1g_ susceptibility yields *γ*_1_ = 0.0825 ± 0.0007, *γ*_2_ = 0.0385 ± 0.0006 with *η* = 0.225 = const, and Δ_0_ = 30.50 meV = const.

The lowest-order contribution to the equilibrium Higgs response is given by the Feynman diagram shown in Fig. 4c (SI S.2.5)^4,47^. With the established parameters *γ*_1_, *γ*_2_, *η*, and Δ_0_ we evaluate the *A*_1g_ response of the Higgs excitation following eq. S21 and show the resulting susceptibility together with the NEARS Higgs feature at 75 μJ cm^−2^ in Fig. 4c. The position of the Higgs excitation within the weak-coupling BCS model relative to 2Δ depends on the symmetry of the order parameter as well as on *r* = *b*_1_/(*b*_0_ + *b*_1_)^50^. Increasing the parameter *r* shifts the peak of the Higgs response towards lower energies. The solid red line in Fig. 4c shows the calculated Higgs response for *r* = 0.88 and *γ*_0_ = 0.071. However, Bi-2212 is a strong-coupling superconductor that goes beyond the BCS weak-coupling model. Moreover, Fig. 4c represents a non-equilibrium state that cannot be adequately captured by an equilibrium calculation. In an equilibrium calculation performed in the clean limit, we find that the Higgs susceptibility is approximately three orders of magnitude weaker than the PB peak. The NEARS experiment, on the other hand, probes the Higgs modes following population inversion in a non-equilibrium state.

Important conclusions can be drawn from our calculations. Figure 4a, b show an excellent agreement between the microscopic theory and our phonon-subtracted electronic equilibrium susceptibilities. This is remarkable, since there is no *A*_1g_ problem^34,51^ in our data taken with an incident photon energy of 3.1 eV. In addition, the Higgs mode is an in-gap excitation that can appear at energies below 2Δ depending on the breaking of particle-hole symmetry.

In conclusion, by introducing NEARS, we find new in-gap excitations in a high-T_C_ superconductor which can be assigned to the Higgs modes. This technique goes beyond conventional Raman spectroscopy for the identification of the symmetry of SC order parameters. NEARS experiments determine energy and life time enabling a first quantitative approach to the dynamics of Higgs modes in a superconductor. NEARS makes Higgs spectroscopy applicable to many materials classes characterized by the interplay of superconductivity and competing or coexisting orders^52–54^. Higgs spectroscopy depends on both the symmetry of the quench and the symmetry of the Raman polarization probing the condensate. The energy and symmetry properties of the SC Higgs field are material-dependent and enable to study and control of Higgs physics. Since the BCS-like Meissner effect requires the presence of the Higgs field, the observation of a Higgs mode can serve as a novel criterion for superconductivity.

## Methods

### Raman instrument

Spontaneous Raman measurements were performed on the UT-3 Raman spectrometer (see Fig. S1)^46^. This triple-grated spectrometer is fully achromatic due to the use of reflective optics. Excellent stray light rejection is achieved via an entrance objective with a large numerical aperture of 0.5 in a Cassegrain-type design, small focal points due to aberration-free off-axis paraboloids in combination with bilateral slits of the pre-monochromator, and an additional relay-stage equipped with two monolateral slits for setting an asymmetric bandpass for Stokes- and anti-Stokes measurements. A beam block in the entrance objective blocks reflected and emitted light in an angle of 21.8° to the vertical (see Fig. S2). In this way, a low-frequency cutoff of less than 5 cm^−1^ can be achieved^46^. However, in the current experiment, the low-frequency cutoff is limited to the natural Fourier-broadening of the laser line of the pulsed laser source to approx. 80 cm^−1^. We utilized a pulsed Ti:Sapphire laser system, Tsunami model HP fs 15 WP (Spectra Physics Lasers Inc., California) at a fundamental wavelength of 802 nm with a second harmonic generation (SHG) unit generating the probe wavelength of 401 nm. The pulse duration was 1.2 ± 0.1 ps monitored with an autocorrelator (APE GmbH, Berlin, Germany). The 401 nm and 802 nm beams were guided over two separate beam paths as shown in Figure S1. For time delay scans, a motorized delay line in the pump beam path was used. A *λ*/2 waveplate in the probe beam path allows symmetry-dependent studies. We have employed *A*_2g_+*B*_1g_ polarization by using crossed polarization and *A*_1g_+*B*_2g_ symmetry by parallel polarization between incident and scattered light with respect to the a and b axes in the CuO_2_ planes. By rotating the sample by 45° the *A*_2g_+*B*_2g_ signal was measured for reference (see Fig. S10) showing that contributions from *A*_2g_ and *B*_2g_ are small. Neutral density filter units/wheels were used for fluence dependence. Figure S2 shows the details of the entrance objective consisting of four on-axis parabolic mirrors, which focuses the light into the first monochromator. Pump and probe beam reach the sample at an angle of 21.8°. By this, we apply a non-zero in-plane momentum to the sample, which causes symmetry breaking and activation of the Higgs mode in *B*_1g_ symmetry.

### Transient Stokes and anti-Stokes Raman measurements

To characterize the beam spot size and shape and align the spatial overlap of the probe and the pump spot, a DFK 23GM021 industrial camera (The Imaging Source, Bremen, Germany) with a pixel size of 3.75 μm was positioned at the focal point of the entrance objective of the UT-3. We used a probe spot (401 nm) of FWHM = 19.5 μm x 11.1 μm (hor x ver) and a pump spot of FWHM = 22.5 μm x 16.9 μm (hor x ver). The applied probe power was 4.75  ± 0.15 mW resulting in a fluence of 35.08 ± 1.15 μJ cm^−2^ at a repetition rate of 80 MHz of the Tsunami system. For the pump, fluences of 20 μJ cm^−2^ to 113 μJ cm^−2^ were used (see Table S2 for details). In order to establish temporal overlap of the two pulses, an ultrafast diode UPD-50-UP (Alphalas, Göttingen, Germany) was placed in the focal point of the entrance objective, where probe and pump beam were focused. A Picoscope 6402B by Pico Technology (Cambridgeshire, United Kingdom) was used to monitor the pulses and find rough temporal overlap. We then conducted reference measurements on highly oriented pyrolytic graphite (HOPG) by Alfa Aesar, Thermo Fisher Scientific (Massachusetts, USA), to calibrate the delay line. Probe-only, pump-probe, pump-only, and background measurements were taken one after the other with an integration time of 30 min each. In general, we conducted 3 repetitions per measurement to improve the signal-to-noise ratio and ensure stability over the measurement time. Stokes and anti-Stokes measurements were carried out with two different settings of the spectrometer bandpass and monolateral slits.

### Sample

The investigated sample is an Y_0.08_-substituted Bi-2212 crystal with a T_c_ of 92 K (see Fig. S3) with the nominal composition Bi_2.00_Sr_2.00_Ca_0.92_Y_0.08_Cu_2_O_8+*δ*_. It is slightly underdoped as compared to ${{{\rm{T}}}}_{{{\rm{c}}},\max }$ = 96 K. The sample was annealed at 500 °C in Argon. For more details on sample growth see previous work^38^. A continuous flow LHe Konti-Cryostat Spectro (CryoVac, Troisdorf, Germany) was utilized to cool the sample down to 8 K base temperature. The used cryostat is an exchange-gas cryostat with active cooling from the side of the laser impinging on the sample. At a typical optical penetration depth of 20 nm - 100 nm, this leads to an effectively base-temperature independent laser heating.

### Data treatment

Raman spectra have been corrected for the static background detector signal and the spectral response of the spectrometer. The data was then normalized to the respective probe laser power and integration time. For NEARS data, no Bose-function correction was applied to the data itself, since the division Bose factor for anti-Stokes spectra converges to zero (see Figure S4). We, therefore, plot the Raman intensity instead of the Raman susceptibility (see Figs. 1, 2 in the main text). However, by utilizing the linkage of Stokes and anti-Stokes data via the Bose-function (see equation S29), one can calculate the anti-Stokes spectrum from the measured Stokes spectrum, and analyze the difference between the calculated anti-Stokes and the measured anti-Stokes data. As a key result of this work, we find no difference signal for all non-pumped data and pump-probe data above T_c_ (see Fig. 1 in the main text). However, in the superconducting state and in the pump-probe measurement, we obtain a difference signal in the anti-Stokes data, which can be attributed to an overpopulation of the excited Higgs state. NEARS maps are derived from the data by plotting the superposition of the Raman susceptibility of the in-gap NEARS feature obtained from anti-Stokes data together with the Raman susceptibility of the PB feature (Stokes side) in an interpolation 2D color plot as a function of excitation energy (see also parameterization method of the PB feature in SI S.6). For this, we utilize the python class scipy.interpolate.interp2d. In order to make this new representation of non-equilibrium Raman data more accessible to the reader, Figure S7 shows the Raman susceptibility of the NEARS feature on an energy-gain (anti-Stokes) axis, together with the superconductivity induced Raman susceptibility on the Stokes side (PB peak) on an energy-loss axis. Raman spectra (Figs. 1, 2) were fitted using the Levenberg-Marquardt fitting routine of Igor Pro (Version 6.3). To fit the electonic Raman response within our BCS model (Fig. 4), we use a Trust Region Reflective (trf) algorithm (scipy.optimize.least_squares).

### Phenomenological model and BCS theory

A phenomenological Ginzburg-Landau model can describe the charged bosonic condensate by using a Klein-Gordon like Lagrangian with a Ginzburg-Landau Mexican-hat potential $F(\Psi )=\alpha | \Psi {| }^{2}+\frac{\beta }{2}| \Psi {| }^{4}$ (*α* < 0) and for small fluctuations of the Higgs amplitude (*H*) around the ground state $| {\Psi }_{0}|=\sqrt{\frac{-\alpha }{\beta }}$ ^47^.

The Higgs mode, characterizing the low-energy excitation spectrum of the condensate, is Raman active and couples quadratically to the vector potential. We can calculate the equation of motion for the Higgs mode by using the Euler-Lagrange equations in the *q* → 0 limit (see SI S.4). In order to account for the non-equilibrium experimental conditions, we can quench the order parameter within the Ginzburg-Landau theory by quenching *α*, *β*, or both of them, since *Ψ*_0_ itself depends on the ratio of $\sqrt{\frac{| \alpha | }{\beta }}$. If we quench *α* we will change the frequency of the Higgs mode to lower energy. As shown in Fig. 2f, we do not observe this behavior in the experiment. If we quench *β*, we will reduce the superfluid density and not change the frequency of the Higgs mode. This case fits our experimental observations. We can calculate the Green’s function in the **q** → 0 case by assuming a *δ* function-like quench due to a change in *β*. See SI S.4 for more details.

We further compare the experimental data to a microscopic BCS weak-coupling theory. A weak-coupling Hamiltonian is utilized

M1 $${{\mathcal{H}}}(t)=\mathop{\sum}\limits_{{{\boldsymbol{k}}},\sigma }{\xi }_{{{\boldsymbol{k}}}-{{\bf{A}}}(t)}{c}_{{{\boldsymbol{k}}},\sigma }^{{\dagger} }{c}_{{{\boldsymbol{k}}},\sigma }-\mathop{\sum}\limits_{{{\boldsymbol{k}}},{{{\boldsymbol{k}}}}^{{\prime} }}{V}_{{{\boldsymbol{k}}},{{{\boldsymbol{k}}}}^{{\prime} }}{c}_{{{\boldsymbol{k}}},\uparrow }^{{\dagger} }{c}_{-{{\boldsymbol{k}}},\downarrow }^{{\dagger} }{c}_{-{{{\boldsymbol{k}}}}^{{\prime} },\downarrow }{c}_{{{{\boldsymbol{k}}}}^{{\prime} },\uparrow }\,.$$

The electron dispersion *ξ*_***k***_ = *ϵ*_***k***_ − *ϵ*_*F*_ is measured relative to the Fermi level and ${c}_{{{\boldsymbol{k}}},\sigma }^{{\dagger} }$ and *c*_***k***,*σ*_ represent the electron creation or annihilation operators. A separable pairing interaction ${V}_{{{\boldsymbol{k}}},{{{\boldsymbol{k}}}}^{{\prime} }}=V{f}_{{{\boldsymbol{k}}}\;}{f}_{{{{\boldsymbol{k}}}}^{{\prime} }}$ with strength *V* and symmetry function *f*_*k*_ is used. The coupling to light is obtained by the expansion of the minimal coupling up to second order in ***A***

M2 $${\xi }_{{{\boldsymbol{k}}}-{{\boldsymbol{A}}}(t)}={\xi }_{{{\boldsymbol{k}}}}-\mathop{\sum}\limits_{i}{\partial }_{i}{\xi }_{{{\boldsymbol{k}}}}{A}_{i}(t)+\frac{1}{2}\mathop{\sum}\limits_{i,j}{\partial }_{ij}^{2}{\xi }_{{{\boldsymbol{k}}}}{A}_{i}(t){A}_{j}(t)+{{\mathcal{O}}}(A{(t)}^{3})\,.$$

The pair-breaking Raman vertices for *B*_1g_ and *A*_1g_ can be expressed as ${\gamma }_{{B}_{1{{\rm{g}}}}}={\gamma }_{b}\cos (2\phi )$ and ${\gamma }_{{A}_{1{{\rm{g}}}}}=(1+{b}_{0}\xi )({\gamma }_{0}+{\gamma }_{1}\cos (4\phi )+{\gamma }_{2}\cos (8\phi ))$, respectively, as outlined in the main text^34^.

The lowest order contribution to the *B*_1g_ pair-breaking susceptibility is not Coulomb-screened^34^ and can be diagrammatically represented as shown in Fig. 4a. The information about the light-matter interaction and the symmetry channel, in particular, is entirely contained in the Raman vertex function, ${\gamma }_{{B}_{1{{\rm{g}}}}}{\sigma }_{3}$. With the dimensionless frequency *x* = *ω*/2Δ_0_ + *iη* we can write algebraically

M3 $${\chi }_{{B}_{1{{\rm{g}}}}{B}_{1{{\rm{g}}}}}({{\boldsymbol{q}}}=0,x)/{N}_{F}=\left\langle {\gamma }_{{B}_{1{{\rm{g}}}}}^{2}\right\rangle={\gamma }_{b}^{2}{I}_{2}(x)\,,$$

with the integrals *I*_*n*_(*x*) defined as

M4 $$\left\langle {\cos }^{2(n-1)}(2\phi )\right\rangle=	{N}_{F}\int_{-{\xi }_{D}}^{{\xi }_{D}}d\xi \int_{0}^{2\pi }d\phi \frac{4{\Delta }_{{{\bf{{k}}}}}^{2}{\cos }^{2(n-1)}(2\phi )}{{E}_{{{\bf{{k}}}}}(4{E}_{{{\bf{{k}}}}}^{2}-{(i{\omega }_{n})}^{2})}\\=	2{N}_{F}\int_{0}^{2\pi }d\phi \frac{{f}_{{{\bf{{k}}}}}^{2n}/{x}^{2}}{\sqrt{{f}_{{{\bf{{k}}}}}^{2}/{x}^{2}-1}}{\tan }^{-1}\left(\frac{x}{\sqrt{{f}_{{{\bf{{k}}}}}^{2}-{x}^{2}}}\right)\\=	4{N}_{F}\int_{0}^{1}dt\frac{{t}^{2n}/{x}^{2}}{\sqrt{1-{t}^{2}}\sqrt{1-{t}^{2}/{x}^{2}}}{{\mathcal{D}}}(x,t):={I}_{n}(x)\\ \,{{\mbox{with}}}\,\quad {{\mathcal{D}}}(x,t)=	\left(\,{{\mbox{sgn}}}\,({{\rm{Re}}} (x))i\pi+\ln \left[\frac{1-\sqrt{1-{t}^{2}/{x}^{2}}}{1+\sqrt{1-{t}^{2}/{x}^{2}}}\right]\right)\,.$$

The *A*_1g_ response is modified by Coulomb screening of the charge fluctuations. The algebraic expression for the screened susceptibility,

M5 $${\chi }_{{A}_{1{{\rm{g}}}}{A}_{1{{\rm{g}}}}}^{sc}={\chi }_{{A}_{1{{\rm{g}}}}{A}_{1{{\rm{g}}}}}-\frac{{\chi }_{{A}_{1{{\rm{g}}}}{\sigma }_{3}}{\chi }_{{\sigma }_{3}{A}_{1{{\rm{g}}}}}}{{\chi }_{{\sigma }_{3}{\sigma }_{3}}}\,,$$

can be expressed after Fermi-surface harmonics expansion as

M6 $${\chi }_{{A}_{1{{\rm{g}}}}{A}_{1{{\rm{g}}}}}^{\,{\rm{sc}}\,}=	64{\gamma }_{2}^{2}{I}_{5}+(32{\gamma }_{1}{\gamma }_{2}-128{\gamma }_{2}^{2}){I}_{4}+(4{\gamma }_{1}^{2}-48{\gamma }_{1}{\gamma }_{2}+80{\gamma }_{2}^{2}){I}_{3}\\ 	+(-4{\gamma }_{1}^{2}+20{\gamma }_{1}{\gamma }_{2}-16{\gamma }_{2}^{2}){I}_{2}+({\gamma }_{1}^{2}-2{\gamma }_{1}{\gamma }_{2}+{\gamma }_{2}^{2}){I}_{1}\\ 	 -\frac{{\left(8{\gamma }_{2}{I}_{3}+(2{\gamma }_{1}-8{\gamma }_{2}){I}_{2}-{\gamma }_{1}{I}_{1}\right)}^{2}}{{I}_{1}}\,.$$

The peak position of the pair-breaking excitation in *A*_1g_ symmetry is strongly dependent on the admixture of the higher-order Fermi-surface harmonics *γ*_1_, *γ*_2_.

The lowest-order contribution to the Higgs response is given by the Feynman diagram shown in Fig. 4c^4,47^. The homogeneous Higgs propagator, assuming an isotropic Fermi-surface and *T* → 0 can be expressed as

M7 $${H}^{-1}(i{\omega }_{n},{{\boldsymbol{q}}}=0)=\mathop{\sum}\limits_{{\boldsymbol{k}}}\frac{{f}_{{\boldsymbol{k}}}^{2}(4{\Delta }_{{\boldsymbol{k}}}^{2}-{i{\omega }_{n}}^{2})}{{E}_{{{\boldsymbol{k}}}}(4{E}_{{{\boldsymbol{k}}}}^{2}-{i{\omega }_{n}}^{2})}\tanh (\beta {E}_{{{\boldsymbol{k}}}}/2)\,.$$

With the established parameters *γ*_1_, *γ*_2_, *η*, and Δ_0_ we can evaluate the *A*_1g_ response of the Higgs excitation following eq. S21. Details of our calculations can be found in the SI (S.2).

## Supplementary information

Supplementary Information

Transparent Peer Review file

## Data Availability

The NEARS data generated in this study have been deposited in the UHH repository (https://www.fdr.uni-hamburg.de/), see ref. ^55^.

## Code availability

Code is available at codeocean.com^56^.

## References

[1] Sooryakumar, R. & Klein, M. Raman scattering by superconducting-gap excitations and their coupling to charge-density waves. *Phys. Rev. Lett.* **45**, 660–662 (1980).

[2] Higgs, P. Prehistory of the Higgs boson. *Comptes Rendus Physique* **8**, 970–972 (2007).

[3] Feng, L. et al. Dynamical interplay between superconductivity and charge density waves: a nonlinear terahertz study of coherently driven 2H-NbSe_2_. *Phys. Rev. B* **108**, L100504 (2023).

[4] Cea, T. & Benfatto, L. Nature and Raman signatures of the Higgs amplitude mode in the coexisting superconducting and charge-density-wave state. *Phys. Rev. B* **90**, 224515 (2014).

[5] Littlewood, P. & Varma, C. Amplitude collective modes in superconductors and their coupling to charge-density waves. *Phys. Rev. B* **26**, 4883–4893 (1982).

[6] Méasson, M. et al. Amplitude Higgs mode in the 2H-NbSe_2_ superconductor. *Phys. Rev. B* **89**, 060503 (2014).

[7] Grasset, R. et al. Higgs-mode radiance and charge-density-wave order in 2H-NbSe_2_. *Phys. Rev. B* **97**, 094502 (2018).

[8] Chu, H. et al. Fano interference between collective modes in cuprate high-T_*C*_ superconductors. *Nat Commun* **14**, 1343 (2023). 36906577 10.1038/s41467-023-36787-4 PMC10008591

[9] Matsunaga, R. et al. Light-induced collective pseudospin precession resonating with Higgs mode in a superconductor. *Science* **345**, 1145–1149 (2014). 25011555 10.1126/science.1254697

[10] Budelmann, D. et al. Gaplike excitations in the superconducting state of Bi_2_Sr_2_CaCu_2_O_8_ studied by resonant Raman scattering. *Phys. Rev. Lett.* **95**, 057003 (2005). 16090910 10.1103/PhysRevLett.95.057003

[11] Saichu, R. et al. Two-component dynamics of the order parameter of high-temperature Bi_2_Sr_2_CaCu_2_O_8+*δ*_ superconductors revealed by time-resolved Raman scattering. *Phys. Rev. Lett.* **102**, 177004 (2009). 19518817 10.1103/PhysRevLett.102.177004

[12] Pellatz, N. et al. Relaxation timescales and electron-phonon coupling in optically pumped YBa_2_Cu_3_O_6+*x*_ reveraled by time-resolved Raman scattering. *Phys. Rev. B* **104**, L180505 (2021).

[13] Han, S. et al. Incoherent phonon population and exciton-exciton annihilation dynamics in monolayer WS_2_ revealed by time-resolved Resonance Raman scattering. *Optics Express* **27**, 29949 (2019). 31684250 10.1364/OE.27.029949

[14] Katsumi, K. et al. Higgs mode in the d-wave superconductor Bi_2_Sr_2_CaCu_2_O_8+*x*_ driven by an intense Terahertz pulse. *Phys. Rev. Lett.* **120**, 117001 (2018). 29601772 10.1103/PhysRevLett.120.117001

[15] Chu, H. et al. Phase-resolved Higgs response in superconducting cuprates. *Nat Commun* **11**, 1793 (2020). 32286291 10.1038/s41467-020-15613-1 PMC7156672

[16] Vaswani, C. et al. Light quantum control of persisting Higgs modes in iron-based superconductors. *Nat Commun* **12**, 258 (2021). 33431843 10.1038/s41467-020-20350-6 PMC7801641

[17] Wang, Z. et al. Transient Higgs oscillations and high-order nonlinear light-Higgs coupling in a terahertz wave-driven NbN superconductor. *Phys. Rev. B* **105**, L100508 (2022).

[18] Reinhoffer, C. et al. High-order nonlinear terahertz probing of the two-band superconductor MgB_2_: third- and fifth-order harmonic generation. *Phys. Rev. B* **106**, 214514 (2022).

[19] Katsumi, K. et al. Revealing novel aspects of light-matter coupling by terahertz two-dimensional coherent spectroscopy: the case of the amplitude mode in superconductors. *Phys. Rev. Lett.* **132**, 256903 (2024). 38996273 10.1103/PhysRevLett.132.256903

[20] Shimano, R. & Tsuji, N. Higgs Mode in Superconductors. *Annu. Rev. Condens. Matter Phys.* **11**, 103–124 (2020).

[21] Kim, M. et al. Tracing the dynamics of superconducting order via transient terahertz third-harmonic generation. *Science Advances* **10**, eadi7598 (2024). 38489363 10.1126/sciadv.adi7598 PMC10942118

[22] Barlas, Y. & Varma, C. Amplitude or Higgs modes in d-wave superconductors. *Phys. Rev. B* **87**, 054503 (2013).

[23] Schwarz, L. & Manske, D. Theory of driven Higgs oscillations and third-harmonic generation in unconventional superconductors. *Phys. Rev. B* **101**, 184519 (2020).

[24] Tsuji, N. & Aoki, H. Theory of Anderson pseudospin resonance with Higgs mode in superconductors. *Phys. Rev. B* **92**, 064508 (2015).

[25] Hannibal, S. et al. Quench dynamics of an ultracold Fermi gas in the BCS regime: Spectral properties and confinement-induced breakdown of the Higgs mode. *Phys. Rev. A* **91**, 043630 (2015).

[26] Benfatto, L., Castellani, C. & Seibold, G. Linear and nonlinear current response in disordered d-wave superconductors. *Phys. Rev. B* **108**, 134508 (2023).

[27] Seibold, G., Udina, M., Castellani, C. & Benfatto, L. Third harmonic generation from collective modes in disordered superconductors. *Phys. Rev. B* **103**, 014512 (2021).

[28] Udina, M. et al. THz non-linear optical response in cuprates: predominance of the BCS response over the Higgs mode. *Faraday Discussions* **237**, 168–185 (2022). 35642718 10.1039/d2fd00016d

[29] Sun, Z., Fogler, M., Basov, D. & Millis, A. Collective modes and terahertz near-field response of superconductors. *Phys. Rev. Res.* **2**, 023413 (2020).

[30] Gabriele, F., Udina, M. & Benfatto, L. Non-linear Terahertz driving of plasma waves in layered cuprates. *Nat Commun* **12**, 752 (2021). 33531492 10.1038/s41467-021-21041-6 PMC7854596

[31] Sellati, N., Gabriele, F., Castellani, C. & Benfatto, L. Generalized Josephson plasmons in bilayer superconductors. *Phys. Rev. B* **108**, 014503 (2023).

[32] Schwarz, L. et al. Classification and characterization of nonequilibrium Higgs modes in unconventional superconductors. *Nat Commun* **11**, 287 (2020). 31941881 10.1038/s41467-019-13763-5 PMC6962398

[33] Munnikes, N. et al. Pair breaking versus symmetry breaking: origin of the Raman modes in superconducting cuprates. *Phys. Rev. B* **84**, 144523 (2011).

[34] Devereaux, T. & Einzel, D. Electronic Raman scattering in superconductors as a probe of anisotropic electron pairing. *Phys. Rev. B* **51**, 16336–16357 (1995). 10.1103/physrevb.51.16336 9978618

[35] Blumberg, G. et al. Observation of Leggett’s collective mode in a multiband MgB_2_ superconductor. *Phys. Rev. Lett.* **99**, 227002 (2007). 18233316 10.1103/PhysRevLett.99.227002

[36] Maiti, S., Maier, T., Böhm, T., Hackl, R. & Hirschfeld, P. Probing the pairing interaction and multiple Bardasis-Schrieffer modes using Raman spectroscopy. *Phys. Rev. Lett.* **117**, 257001 (2016). 28036201 10.1103/PhysRevLett.117.257001

[37] Böhm, T. et al. Microscopic origin of Cooper pairing in the iron-based superconductor Ba_1-*x*_K_*x*_Fe_2_As_2_. *Npj Quant Mater.* **3**, 48 (2018).

[38] Eisaki, H. et al. Effect of chemical inhomogeneity in bismuth-based copper oxide superconductors. *Phys. Rev. B* **69**, 064512 (2004).

[39] Bock, A. Laser heating of YBa_2_Cu_3_O_7_ films in Raman experiments. *Phys. Rev. B* **51**, 15506–15518 (1995). 10.1103/physrevb.51.15506 9978510

[40] Klein, M. et al. Resonance Raman study of 2Δ-gap like features in superconducting Bi-2212 and YBCO. *J. Phys. Chem. Solids* **67**, 298 - 301 (2006).

[41] Perfetti, L. et al. Ultrafast electron relaxation in superconducting Bi_2_Sr_2_CaCu_2_O_8+*δ*_ by time-resolved photoelectron spectroscopy. *Phys. Rev. Lett.* **99**, 197001 (2007). 18233106 10.1103/PhysRevLett.99.197001

[42] Graf, J. et al. Nodal quasiparticle meltdown in ultrahigh-resolution pump-probe angle-resolved photoemission. *Nat. Phys.* **7**, 805–809 (2011).

[43] Smallwood, C. et al. Tracking Cooper pairs in a cuprate superconductor by ultrafast angle-resolved photoemission. *Science* **336**, 1137–1139 (2012). 22654053 10.1126/science.1217423

[44] Toda, Y. et al. Rotational symmetry breaking in Bi_2_Sr_2_CaCu_2_O_8+*δ*_ probed by polarized femtosecond spectroscopy. *Phys. Rev. B* **90**, 094513 (2014).

[45] Henry, B. & Kasha, M. Radiationless molecular electronic transitions. *Annu. Rev. Phys. Chem.* **19**, 161–192 (1968).

[46] Schulz, B. et al. Fully reflective deep ultraviolet to near infrared spectrometer and entrance optics for resonance Raman spectroscopy. *Rev. Sci. Instrum.* **76**, 073107 (2005).

[47] Puviani, M., Schwarz, L., Zhang, X., Kaiser, S. & Manske, D. Current-assisted Raman activation of the Higgs mode in superconductors. *Phys. Rev. B* **101**, 220507 (2020).

[48] Legros, A. et al. Universal T-linear resistivity and Planckian dissipation in overdoped cuprates. *Nat. Phys.* **15**, 142–147 (2019).

[49] Hwang, J. Superconducting coherence length of hole-doped cuprates obtained from electron-boson spectral density function. *Sci. Rep.* **11**, 11668 (2021). 34083645 10.1038/s41598-021-91163-w PMC8175355

[50] Cea, T., Castellani, C. & Benfatto, L. Nonlinear optical effects and third-harmonic generation in superconductors: cooper pairs versus Higgs mode contribution. *Phys. Rev. B* **93**, 180507 (2016).

[51] Krantz, M. & Cardona, M. Raman scattering by electronic excitations in semiconductors and in high T_*C*_ superconductors. *J. Low Temp. Phys.* **99**, 205–221 (1995).

[52] Bardasis, A. & Schrieffer, J. Excitons and plasmons in superconductors. *Phys. Rev.* **121**, 1050–1062 (1961).

[53] Norman, M. The challenge of unconventional superconductivity. *Science* **332**, 196–200 (2011). 21474750 10.1126/science.1200181

[54] Service, R. At last, room temperature superconductivity achieved. *Science* **370**, 273–274 (2020). 33060340 10.1126/science.370.6514.273

[55] Glier, T. et al. Data set: non-equilibrium anti-Stokes Raman spectroscopy for investigating Higgs modes in superconductors. *UHH Respository*. 10.25592/uhhfdm.14834 (2025). 10.1038/s41467-025-62245-4 PMC12314123 40744925

[56] Tian, S., Dolgner, J., Glier, T., Rübhausen, M. & Manske, D. Non-Equilibrium Anti-Stokes Raman Spectroscopy for Investigating Higgs Modes in Superconductors [Source Code], 10.24433/CO.6806705.v1 (2025). 10.1038/s41467-025-62245-4 PMC12314123 40744925

