# Supplementary material for: Non-equilibrium anti-Stokes Raman spectroscopy for investigating Higgs modes in superconductors
Source: Nat Commun. 2025 Jul 31;16:7027. doi: 10.1038/s41467-025-62245-4 (PMC12314123; doi:10.1038/s41467-025-62245-4)
Supplement: Supplementary file 1 — Supplementary Information [file 41467_2025_62245_MOESM1_ESM.pdf]

Non-Equilibrium Anti-Stokes Raman Spectroscopy for Investigating Higgs Modes in Superconductors

Tomke E. Glier[1*], Sida Tian[2], Mika Rerrer[1], Lea Westphal[1,†], Garret Lüllau[1,‡], Liwen Feng[3], Jakob Dolgner[2], Rafael Haenel[2], Marta Zonno[2,4,5,§], Hiroshi Eisaki[6], Martin Greven[7], Andrea Damascelli[4,5], Stefan Kaiser[3*], Dirk Manske[2*], Michael Rübhausen[1*]

[1]Institute of Nanostructure and Solid State Physics, Universität Hamburg, Hamburg, 22761, Germany.
[2]Max Planck Institute for Solid State Research, Stuttgart, 70569, Germany.
[3]Institute of Solid State and Materials Physics, TUD Dresden University of Technology, Dresden, 01062, Germany.
[4]Quantum Matter Institute, University of British Columbia, Vancouver, BC V6T 1Z4, Canada.
[5]Department of Physics & Astronomy, University of British Columbia, Vancouver, BC V6T 1Z1, Canada.
[6]Nanoelectronics Research Institute, National Institute of Advanced Industrial Science and Technology, Tsukuba, Ibaraki, 305-8568, Japan.
[7]School of Physics and Astronomy, University of Minnesota, Minneapolis, MN 55455, USA.
[†]Present Address: Heinz Maier-Leibnitz Zentrum (MLZ), Technische Universität München, Garching, 85748, Germany.
[‡]Present Address: Laboratoire MPQ, Université Paris Cité, Paris, 75013, France.
[§]Present Adress: Synchrotron SOLEIL, Saint-Aubin, 91190, France.

*Corresponding author(s). E-mail(s): tomke.glier@uni-hamburg.de; stefan.kaiser@tu-dresden.de; d.manske@fkf.mpg.de; michael.ruebhausen@uni-hamburg.de;

Supplementary Information

S.1 BCS-Dirac Correspondence

In the following, we elaborate on an analogy between the BCS- and Dirac Hamiltonian, which is repeatedly pointed out in the literature.[1, 2]

In natural units, the Dirac Lagrangian of relativistic Fermions in Weyl representation with the gamma matrices

$$\gamma^0 = \begin{pmatrix} 0 & \mathbb{1} \\ \mathbb{1} & 0 \end{pmatrix} \quad \text{and} \quad \gamma^i = \begin{pmatrix} 0 & \sigma^i \\ -\sigma^i & 0 \end{pmatrix} \quad \text{is} \quad \mathcal{L}^{\text{Dirac}} = \bar{\Psi} \left(i\partial\!\!\!/ - m \right) \Psi \,. \tag{S1}$$

Legendre transformation using the canonical momentum $\frac{\delta \mathcal{L}}{\delta \partial_0 \Psi} = i\Psi^\dagger$ results in the Dirac-Hamiltonian[3]

$$\mathcal{H}^{\text{Dirac}} = \Psi^\dagger \left(i\boldsymbol{\sigma} \cdot \boldsymbol{\nabla} \hat{\sigma}_3 + m\hat{\sigma}_1 \right) \Psi = \begin{pmatrix} \Psi_L^\dagger & \Psi_R^\dagger \end{pmatrix} \begin{pmatrix} i\boldsymbol{\sigma} \cdot \boldsymbol{\nabla} & m \\ m & -i\boldsymbol{\sigma} \cdot \boldsymbol{\nabla} \end{pmatrix} \begin{pmatrix} \Psi_L \\ \Psi_R \end{pmatrix} . \tag{S2}$$

Here we use $\hat{\sigma}_i = \sigma_i \otimes \mathbb{1}_{2\text{x}2}$ to distinguish the extension of the Pauli matrices to 4×4 block matrices. The eigenvalues of this Hamiltonian are $E_{\mathbf{p}} = \pm\sqrt{\mathbf{p}^2 c^2 + m^2 c^4}$.

Using Nambu spinor notation the BCS-Hamiltonian (without local $U(1)$ symmetry i.e. uncharged BCS) may be rewritten into a Bogoliubov-deGennes Hamiltonian,

$$\mathcal{H}^{\text{BdG}} = \sum_{\mathbf{k}} \Psi_{\mathbf{k}}^\dagger \left(\xi_{\mathbf{k}} \sigma_3 + \Delta_{\mathbf{k}} \sigma_1 \right) \Psi_{\mathbf{k}} = \sum_{\mathbf{k}} \begin{pmatrix} \Psi_{\mathbf{k},\uparrow}^\dagger & \Psi_{-\mathbf{k},\downarrow} \end{pmatrix} \begin{pmatrix} \xi_{\mathbf{k}} & \Delta_{\mathbf{k}} \\ \Delta_{\mathbf{k}} & -\xi_{\mathbf{k}} \end{pmatrix} \begin{pmatrix} \Psi_{\mathbf{k},\uparrow} \\ \Psi_{-\mathbf{k},\downarrow}^\dagger \end{pmatrix} . \tag{S3}$$

The BdG Hamiltonian's eigenvalues are $E_{\mathbf{k}} = \pm\sqrt{\xi_{\mathbf{k}}^2 + \Delta_{\mathbf{k}}^2}$. Unless the electronic dispersion $\epsilon_{\mathbf{k}}$ is linear in momentum, $E_{\mathbf{k}}$ is not a Lorentz invariant dispersion of the quasiparticles. However, in the vicinity of the Fermi surface $|\xi_{\mathbf{k}}| \ll W$, the bandwidth, we may approximate $\xi_{\mathbf{k}} \approx v_F(|\mathbf{k}| - k_F)$. For s-wave symmetry ($\Delta_{\mathbf{k}} = \Delta$) one can make the analogy explicit by introduction of $m_\Delta = \frac{\Delta}{v_F^2}$ and obtains

$$E_{\mathbf{k}}^{\text{BCS}} = \sqrt{(|\mathbf{k}| - k_F)^2 v_F^2 + m_\Delta^2 v_F^4} \,, \tag{S4}$$

resembling the Dirac Hamiltonian's relativistic dispersion relation with the speed of light replaced by the Fermi velocity v_F.

It is worth mentioning that the (Bogoliubov quasi-)particle-hole symmetry of the BdG-Hamiltonian, in contrast to that of the Dirac Hamiltonian, is only a formal one [4]. Ultimately, this is because in constructing the BdG-Hamiltonian one is doubling the Hilbert space $V \to W = V \oplus V^*$, for which the space of positive energy states $W_+ = V_+ \oplus V_-^*$ and that of negative energy states $W_- = V_- \oplus V_+^*$ are always isomorphic (using the Fréchet-Riesz isomorphism). It is then easy to show, that h^{BdG} satisfies particle-hole symmetry independently of physical properties like the dispersion $\xi_{\mathbf{k}}$, gap Δ or whether $V_+ \simeq V_-$. In particular,

this is also true for $\Delta = 0$. Therefore, this formal particle-hole symmetry is not due to superconductivity and should not be confused with a physical symmetry. On the contrary, it may be viewed as a constraint, enforcing the dependence between the Nambu spinors $\Psi^\dagger$ and Ψ, which a Hamiltonian must satisfy to be a BdG-Hamiltonian [5]. The physical particle-hole symmetry mentioned in the context of superconductors is an approximate symmetry restricted to the states of energies $E \sim \Delta$ close to the Fermi surface. It is essentially just a consequence of Taylor expanding the density of states to zeroth order, which is often a sufficiently good approximation in conventional superconductors because $\Delta \ll W$.

S.2 Electronic Raman Response in a BCS Weak-Coupling Theory

With an energy of 3 eV and an effective bandwidth of only 1 eV in Bi-2212 [6], the probe pulse is resonant. To account for the full Raman cross-section, we would need to consider every band in this energy range (see [7] for a general review of electronic Raman scattering theory). Such a many-band calculation for resonant Raman scattering is outside the scope of this article. Instead, we work with a simple single-band toy model (equation (2) of the main text) capturing the properties of superconductivity, and parametrize the Raman vertices in terms of generalized Fermi surface harmonics, which are solely based on symmetry restrictions. It is assumed that the pairing interaction is restricted to some relatively narrow hull around the Fermi surface, just like in conventional superconductivity. This makes the susceptibilities (S5) less sensitive to the dispersion, which we exploit by replacing it with a simple (but not constant) approximation for the density of states around the Fermi surface. We introduce the expansion coefficients $(\gamma_0, \gamma_1, \gamma_2, \gamma_b, b_0, b_1)$ as fitting parameters, capturing the dispersion and particle-hole asymmetry. Thus, we are able to make statements based entirely on the gap- and Fermi surface symmetries.

Both the pair-breaking and the Higgs response are constructed from common building blocks, the homogeneous polarization/susceptibility diagrams, which we define as

$$\chi_{\Gamma_1,\Gamma_2}(i\omega_n) =$$

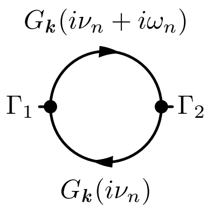

$$= -\frac{1}{\beta}\sum_{\boldsymbol{k},i\nu_n} \mathrm{Tr}[\Gamma_1 G_{\boldsymbol{k}}(i\nu_n + i\omega_n)\Gamma_2 G_{\boldsymbol{k}}(i\nu_n)]\,, \tag{S5}$$

with $i\omega_n = 2n\pi/\beta$ denoting the bosonic and $i\nu_n = (2n+1)\pi/\beta$ the fermionic Matsubara frequencies. We calculate the homogeneous ($\mathbf{q} = 0$) functions because we can safely use the dipole approximation given the probe wavelength of 400 nm and coherence length $\xi \lesssim 2$ nm [8]. The electronic Green's functions of the BCS model written in the basis of Nambu spinors are defined as

$$G_{\boldsymbol{k}}(i\nu_n) = \frac{i\nu_n + \xi_{\boldsymbol{k}}\sigma_3 + \Delta_{\boldsymbol{k}}\sigma_1}{(i\nu_n)^2 - E_{\boldsymbol{k}}^2} = \frac{1}{(i\nu_n)^2 - E_{\boldsymbol{k}}^2}\begin{pmatrix} i\nu_n + \xi_{\boldsymbol{k}} & \Delta_{\boldsymbol{k}} \\ \Delta_{\boldsymbol{k}} & i\nu_n - \xi_{\boldsymbol{k}} \end{pmatrix}, \tag{S6}$$

where $\Delta_{\boldsymbol{k}} = \Delta_0 f_{\boldsymbol{k}}$ and $f_{\boldsymbol{k}} = \cos 2\phi$ for d-wave pairing, $E_{\boldsymbol{k}} = \sqrt{\xi_{\boldsymbol{k}}^2 + \Delta_{\boldsymbol{k}}^2}$ is the Bogoliubov-quasiparticle dispersion and σ_i are the Pauli matrices. Γ_1 and Γ_2 are 2x2-matrix-valued vertices, selecting the appropriate components of the matrix-valued Green's function upon contraction. In this work, there are three types of vertices, $\Gamma_i \in \{f_{\boldsymbol{k}}\sigma_1, \sigma_3, \gamma_{\boldsymbol{k}}\sigma_3\}$ ($f_{\boldsymbol{k}}$ and $\gamma_{\boldsymbol{k}}$ are scalar-valued functions) which are distinguished by different vertex shapes in Feynman diagrams.

For the light-matter coupling, we use the Raman vertex $\gamma_{\boldsymbol{k}}$ which we expand in terms of irreducible representations (irreps) of the lattice symmetry group, e.g. $\gamma_{A_{1g}}$, $\gamma_{B_{1g}}$.

We absorb the matrix structure of the γ vertices to improve readability since it is the same for all irreps and write e.g. $\chi_{A_{1g}\Gamma_2} = \chi_{\gamma_{A_{1g}}\sigma_3,\Gamma_2}$. Similarly, we adapt the notation $\chi_{\Delta\Gamma_2} = \chi_{f_{\boldsymbol{k}}\sigma_1,\Gamma_2}$ and define an expectation value wrt. the Tsuneto function,

$$\langle \alpha(\boldsymbol{k}) \rangle = \sum_{\boldsymbol{k}} \frac{4\Delta_{\boldsymbol{k}}^2 \alpha(\boldsymbol{k})}{E_{\boldsymbol{k}}(4E_{\boldsymbol{k}}^2 - (i\omega_n)^2)} \tanh(\beta E_{\boldsymbol{k}}/2)\,. \tag{S7}$$

This allows us to write compactly,

Table S1: Summary of the elementary susceptibilities which the observables are constructed from.

bare A_{1g} pair breaking response	$\chi_{A_{1g}A_{1g}} = \left\langle (\gamma_{A_{1g}})^2 \right\rangle$
anomalous susceptibility	$\chi_{\Delta\Delta} = \frac{1}{\Delta_0^2} \langle \xi_{\boldsymbol{k}}^2 \rangle$
bare light-Higgs coupling	$\chi_{\Delta A_{1g}} = \chi_{A_{1g}\Delta} = -\frac{1}{\Delta_0} \left\langle \xi_{\boldsymbol{k}} \gamma_{A_{1g}} \right\rangle$
bare charge-fluctuation susceptibility	$\chi_{\sigma_3\sigma_3} = \langle 1 \rangle$
charge-fluctuation-Higgs coupling	$\chi_{\sigma_3\Delta} = \chi_{\Delta\sigma_3} = -\frac{1}{\Delta_0} \langle \xi_{\boldsymbol{k}} \rangle$
light-charge coupling	$\chi_{A_{1g}\sigma_3} = \chi_{\sigma_3 A_{1g}} = \langle \gamma_{A1g} \rangle$

where "bare" refers to the lack of Coulomb screening and A_{1g} may be replaced at any point it appears by another irrep to obtain the respective susceptibility.

S.2.1 Ansatz for Raman vertices and density of states

In the simplified one-band model of the d-wave superconductors, the square lattice symmetry of the cuprates typically leads to Fermi surfaces that are either connected around $\Gamma = (0, 0)$, or $M = (\pm\pi, \pm\pi)$. We choose polar coordinates whose angle ϕ is defined as described in Fig. S14 with respect to the point that the Fermi surface encloses. Following the ansatz proposed in [9], we simplify the Raman vertices by parameterizing their angular dependencies with basis functions in the irreps of the tetragonal D^{4h} point group: $\gamma_{A_{1g}} = \sum_{L=0} \gamma_L^{A_{1g}} \cos(4L\phi)$, $\gamma_{B_{1g}} = \sum_{L=1} \gamma_L^{B_{1g}} \cos([4L-2]\phi)$. We follow previous research [9] by keeping up to $L = 2$ for the A_{1g} response, and only the $L = 1$ term for the B_{1g} response. In this work we lift the

approximate particle-hole symmetry of superconductivity (S.1) by introducing weak energy dependencies, $\gamma_{A1g}(\xi,\phi)$, $N(\xi)$, in the $A_{1\mathrm{g}}$ vertex and the density of states N.

To summarize, the ansatz for the Raman vertices and the density of states is:

$$\begin{aligned} \gamma_{A_{1\mathrm{g}}} &= (1+b_0\xi)(\gamma_0+\gamma_1\cos(4\phi)+\gamma_2\cos(8\phi)) \\ \gamma_{B_{1\mathrm{g}}} &= \gamma_b\cos(2\phi) \\ N(\xi) &= N_F(1+b_1\xi) \end{aligned} \tag{S8}$$

here $\gamma_0,\gamma_1,\gamma_2$ are the coefficients in the generalized Fermi surface harmonic expansion of $\gamma_{A_{1\mathrm{g}}}$, likewise γ_b is the coefficient in the expansion of $\gamma_{B_{1\mathrm{g}}}$. Energy dependence of $\gamma_{A_{1\mathrm{g}}}$ is parametrized by b_0. The deviation from a constant density of state in the thin energy hull around the Fermi surface, inside which we assume the net-attractive electron-electron interaction, is represented by b_1. We assume that the energy dependencies b_0 and b_1 are small $b_0\Delta_0, b_1\Delta_1 \ll 1$. In this parametrization, the average becomes

$$\langle\alpha(\xi(\boldsymbol{k}),\phi(\boldsymbol{k}))\rangle = \int_{-\infty}^{\infty} d\xi \int_0^{2\pi} d\phi \frac{N_F(1+b_1\xi)4\Delta(\phi)^2\alpha(\xi,\phi)}{\sqrt{\xi^2+\Delta(\phi)^2}(4(\xi^2+\Delta(\phi)^2)-(i\omega_n)^2)} \tag{S9}$$

where we dropped the tanh encoding temperature dependence from (S7), whereby we approximate $T=0$ for the electronic degrees of freedom. For the highest pump fluence in the experiment, which we use to extract the Higgs signal (see Fig. 4 main text), the effective quasi-equilibrium temperature due to heating is determined to be $T_{\mathrm{eff}} \approx 98 \pm 13.75\,\mathrm{K}$. The thermal energy $k_BT \approx 8$ meV is still significantly smaller than the lowest energy eigenvalue $E_{\mathbf{k}=0} = \Delta_0 \approx 30$ meV introducing an error of 5% for $E_{\mathbf{k}=0}$ that decays exponentially for higher energy eigenvalues. For the unpumped data, used to fit the pair-breaking peaks, with $T_{\mathrm{eff}} \approx 17\,\mathrm{K}$ (8 K base temperature plus 9 K probe heating (see section S.5)) the zero temperature approximation is even better.

S.2.2 $\mathcal{I}$ and $\mathcal{M}$ functions

We can express the susceptibilities in terms of two classes of integrals, the first of which is given by

$$\begin{aligned} \left\langle\cos^{2(n-1)}(2\phi)\right\rangle &= N_F\int_{-\xi_D}^{\xi_D} d\xi \int_0^{2\pi} d\phi \frac{4\Delta_{\boldsymbol{k}}^2\cos^{2(n-1)}(2\phi)}{E_{\boldsymbol{k}}(4E_{\boldsymbol{k}}^2-(i\omega_n)^2)} \\ &= 2N_F\int_0^{2\pi} d\phi \frac{f_{\boldsymbol{k}}^{2n}/x^2}{\sqrt{f_{\boldsymbol{k}}^2/x^2-1}}\tan^{-1}\left(\frac{x}{\sqrt{f_{\boldsymbol{k}}^2-x^2}}\right) \\ &= 4N_F\int_0^1 dt \frac{t^{2n}/x^2}{\sqrt{1-t^2}\sqrt{1-t^2/x^2}}\mathcal{D}(x,t) := \mathcal{I}_n(x) \\ \text{with}\quad \mathcal{D}(x,t) &= \left(\mathrm{sgn}(\mathrm{Re}(x))i\pi + \ln\left[\frac{1-\sqrt{1-t^2/x^2}}{1+\sqrt{1-t^2/x^2}}\right]\right). \end{aligned} \tag{S10}$$

In the first equality, we inserted the definition (S9) of the expectation value and exploited that the b_1 term vanishes for energy-independent functions in the expectation value. In the second step, we exploit that

$f_{\mathbf{k}} = \cos 2\phi$ and extended $\xi_D \to \infty$, introducing an error $\mathcal{O}\left(\frac{\Delta_0^2}{\xi_D^2}\right)$, to evaluate the energy integral. This is valid in the $\xi_D \gg \Delta_0$ limit. We use $\xi_D/\Delta_0 = 5$. Furthermore, we performed analytical continuation of the frequency and introduced $x = \omega/2\Delta_0 + i\eta$. Finally, we make a substitution $t = \cos(2\phi)$. The sgn(x) function gives the sign of x. The second class of integrals is

$$\mathcal{M}_n(\xi_D, x) \coloneqq \frac{1}{\Delta_0^2}\left\langle \xi^2 \cos^{2(n-1)}(2\phi)\right\rangle . \tag{S11}$$

This function is logarithmically divergent in the energy cut-off ξ_D. Such a divergence exists in the usual BCS-like weak coupling theory and is the consequence of the point-like attractive interaction V. The dependence on the energy cut-off is equivalent to a dependence in the magnitude of the interaction V and is necessary for numerical simulations. We have verified that in the range $\xi_D \gg \Delta_0$ results do not change significantly.

S.2.3 B_{1g} Pair-Breaking Response

The lowest order contribution to the B_{1g} pair-breaking susceptibility is not Coulomb-screened [9] and can be diagrammatically represented by:

$\chi_{B_{1g}B_{1g}} =$ 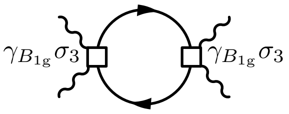 , (S12)

where the wiggly lines symbolize light and the plain lines represent electronic propagators. The information about the light-matter interaction and the symmetry channel, in particular, is entirely contained in the Raman vertex function, $\gamma_{B_{1g}}\sigma_3$. Algebraically,

$$\chi_{B_{1g}B_{1g}}(\boldsymbol{q} = 0, x)/N_F = \left\langle \gamma_{B_{1g}}^2 \right\rangle = \gamma_b^2 \mathcal{I}_2(x) \tag{S13}$$

with the dimensionless frequency $x = \omega/2\Delta_0 + i\eta$.

S.2.4 A_{1g} Pair-Breaking Response

The A_{1g} response is modified by Coulomb screening of the charge fluctuations [10]. These are not present in the B_{1g} response because the Coulomb interaction lies in the A_{1g} channel and therefore cannot couple to B_{1g}. Diagrammatically, the screening shows up as an additional contribution capturing the screened Coulomb interaction between the charge fluctuations due to the Raman probe.

$\chi^{sc}_{A_{1g}A_{1g}} =$ 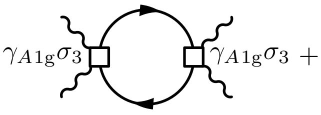 $+$ 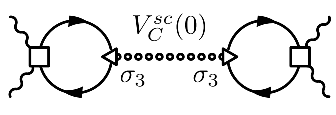 , (S14)

with the screened Coulomb interaction at zero momentum given by $V_C^{sc}(0) = -\chi^{-1}_{\sigma_3\sigma_3}$. The algebraic expression for the screened susceptibility,[10]

$$\chi^{sc}_{A_{1g}A_{1g}} = \chi_{A_{1g}A_{1g}} - \frac{\chi_{A_{1g}\sigma_3}\chi_{\sigma_3 A_{1g}}}{\chi_{\sigma_3\sigma_3}}\,, \tag{S15}$$

becomes lengthy upon Fermi-surface harmonic expansion.

$$\begin{aligned}
\chi_{A_{1g}A_{1g}} &= \big\langle(\gamma_{A_{1g}})^2\big\rangle = \big\langle(1+b_0\xi)^2(\gamma_0+\gamma_1\cos(4\phi)+\gamma_2\cos(8\phi))^2\big\rangle \\
&= 64\gamma_2^2\mathcal{I}_5 + (32\gamma_1\gamma_2 - 128\gamma_2^2)\mathcal{I}_4 + (4\gamma_1^2 + 16\gamma_0\gamma_2 - 48\gamma_1\gamma_2 + 80\gamma_2^2)\mathcal{I}_3 \\
&\quad + (4\gamma_0\gamma_1 - 4\gamma_1^2 - 16\gamma_0\gamma_2 + 20\gamma_1\gamma_2 - 16\gamma_2^2)\mathcal{I}_2 + (\gamma_0^2 - 2\gamma_0\gamma_1 + \gamma_1^2 + 2\gamma_0\gamma_2 - 2\gamma_1\gamma_2 + \gamma_2^2)\mathcal{I}_1 \\
&\quad + (b_0^2 + 2b_0b_1)\Delta_0^2\big\{64\gamma_2^2\mathcal{M}_5 + (32\gamma_1\gamma_2 - 128\gamma_2^2)\mathcal{M}_4 + (4\gamma_1^2 + 16\gamma_0\gamma_2 - 48\gamma_1\gamma_2 + 80\gamma_2^2)\mathcal{M}_3 \\
&\quad + (4\gamma_0\gamma_1 - 4\gamma_1^2 - 16\gamma_0\gamma_2 + 20\gamma_1\gamma_2 - 16\gamma_2^2)\mathcal{M}_2 + (\gamma_0^2 - 2\gamma_0\gamma_1 + \gamma_1^2 + 2\gamma_0\gamma_2 - 2\gamma_1\gamma_2 + \gamma_2^2)\mathcal{M}_1\big\} \\
\chi_{A_{1g}\sigma_3} &= 8\gamma_2\mathcal{I}_3 + (2\gamma_1 - 8\gamma_2)\mathcal{I}_2 + (\gamma_0 - \gamma_1)\mathcal{I}_1 + b_0b_1\Delta_0^2\big\{8\gamma_2\mathcal{M}_3 + (2\gamma_1 - 8\gamma_2)\mathcal{M}_2 + (\gamma_0 - \gamma_1)\mathcal{M}_1\big\} \\
\chi_{\sigma_3\sigma_3} &= \mathcal{I}_1
\end{aligned} \tag{S16}$$

However, all terms containing γ_0 but not b_0 cancel between the bare response and the screening since $\langle\gamma_0 f(\phi)\rangle = \frac{\langle\gamma_0\rangle\langle f(\phi)\rangle}{\langle 1\rangle}$. Because terms linear in ξ vanish in the integral due to symmetry, only terms proportional to b_ib_j with $i,j \in \{0,1\}$ appear. Furthermore, the $\mathcal{M}_i$ terms proportional to b_0 and b_1 are of the same order as the $\mathcal{I}_i$. Since $b_ib_j\Delta_0^2 \ll 1$, the terms due to energy dependence give a negligible contribution to the A_{1g} pair breaking response. Then neglecting the terms proportional to b_ib_j and simplifying the rest results in the more compact expression,

$$\begin{aligned}
\chi^{\mathrm{sc}}_{A_{1g}A_{1g}} &= 64\gamma_2^2\mathcal{I}_5 + (32\gamma_1\gamma_2 - 128\gamma_2^2)\mathcal{I}_4 + (4\gamma_1^2 - 48\gamma_1\gamma_2 + 80\gamma_2^2)\mathcal{I}_3 \\
&\quad + (-4\gamma_1^2 + 20\gamma_1\gamma_2 - 16\gamma_2^2)\mathcal{I}_2 + (\gamma_1^2 - 2\gamma_1\gamma_2 + \gamma_2^2)\mathcal{I}_1 \\
&\quad - \frac{(8\gamma_2\mathcal{I}_3 + (2\gamma_1 - 8\gamma_2)\mathcal{I}_2 - \gamma_1\mathcal{I}_1)^2}{\mathcal{I}_1}\,.
\end{aligned} \tag{S17}$$

The peak position of the pair-breaking excitation in A_{1g} symmetry is strongly dependent on the admixture of the higher-order Fermi-surface harmonics γ_1, γ_2. Experimentally, it is known that the pair-breaking peak changes as a function of the incident photon energy.[11] Changing the ratio between γ_1 and γ_2 accommodates this effect.

S.2.5 Higgs Response

It is well established that the lowest-order contribution to the Higgs response is given by the diagram [12][13]

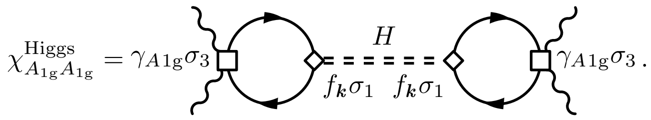

(S18)

The Higgs propagator is mathematically equivalent to the attractive pairing V dressed at RPA level, much like how plasmons can be understood as an RPA-dressed Coulomb interaction. Diagrammatically, it takes the form [14]

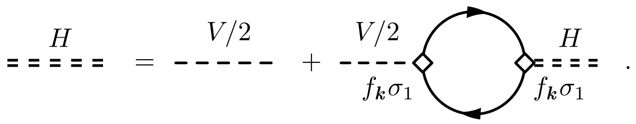

(S19)

The homogeneous Higgs propagator, assuming an isotropic Fermi-surface and at low temperature can be expressed in terms of the integrals $\mathcal{I}_1$ and $\mathcal{I}_2$:

$$\begin{aligned} H^{-1}(i\omega_n, \boldsymbol{q}=0) &= \frac{2}{V} - \chi_{\Delta\Delta} = \sum_{\boldsymbol{k}} \frac{f_{\boldsymbol{k}}^2(4\Delta_{\boldsymbol{k}}^2 - (i\omega_n)^2)}{E_{\boldsymbol{k}}(4E_{\boldsymbol{k}}^2 - (i\omega_n)^2)} \tanh(\beta E_{\boldsymbol{k}}/2) \\ &\overset{T\approx 0}{\approx} N_F \int \frac{t^4/x^2 - t^2}{\sqrt{1-t^2}\sqrt{1-t^2/x^2}} \mathcal{D}(x,t)dt \\ &= \frac{N_F}{4}\left(\mathcal{I}_2 - x^2\mathcal{I}_1\right) \end{aligned} \tag{S20}$$

The density of states introduces an extra $(1 + b_1\xi)$ factor inside all $\langle\cdot\rangle$. Terms odd in ξ vanish under the integral, thus the Higgs propagator is not affected by the introduction of b_1.

The A_{1g} channel of the Higgs response is also affected by Coulomb screening [14]. The *screened Higgs response* $\chi^{\text{Higgs, sc}}_{A_{1g}A_{1g}}$ is obtained by screening both the Higgs propagator and the light-Higgs coupling according to

$$\chi^{\text{Higgs, sc}}_{A_{1g}A_{1g}} = \frac{(\chi_{A_{1g}\Delta} - \chi_{A_{1g}\sigma_3}\chi_{\sigma_3\Delta}/\chi_{\sigma_3\sigma_3})^2}{H^{-1} + \chi_{\Delta\sigma_3}\chi_{\sigma_3\Delta}/\chi_{\sigma_3\sigma_3}} = (\chi^{\text{sc}}_{A_{1g}\Delta})^2 H^{\text{sc}}\,, \tag{S21}$$

with

$$\begin{aligned} \chi_{A_{1g}\Delta} &= -\frac{1}{\Delta_0}\left\langle\gamma_{A_{1g}}\xi\right\rangle \\ &= -\frac{1}{\Delta_0}\left\langle(1+b_0\xi)(\gamma_0 + \gamma_1\cos(4\phi) + \gamma_2\cos(8\phi))\xi\right\rangle \\ &= -\Delta_0(b_0+b_1)\Big([\gamma_0 - \gamma_1 + \gamma_2]\mathcal{M}_1 + [2\gamma_1 - 8\gamma_2]\mathcal{M}_2 + 8\gamma_2\mathcal{M}_3\Big) \\ \chi_{\sigma_3\Delta} &= -\frac{1}{\Delta_0}\langle\xi\rangle = -b_1\Delta_0\mathcal{M}_1\,. \end{aligned} \tag{S22}$$

As can be seen above the light-Higgs coupling, $\chi_{A_{1g}\Delta}$ scales with the sum $b_0 + b_1$. However, unlike b_0 which is a property only of the Raman vertex, b_1 measures the deviation from a constant density of states and thus the breaking of particle-hole symmetry. Charge fluctuations will couple to the pairing channel, allowing screening of the Higgs mode by the background charges. Notice that in (S22) the coupling of the Higgs mode to charge fluctuations $\chi_{\sigma_3\Delta}$, which leads to Coulomb screening, scales only with b_1. We introduce a parameter $r = b_1/(b_0 + b_1)$, controlling the ratio between Coulomb screening and light coupling of the Higgs mode. Increasing the parameter r shifts the peak of the full Higgs response towards smaller energies.

S.2.6 Determining γ and b parameters

In the above approximation, information of the band structure $\xi_{\boldsymbol{k}}$ is encoded in the Raman vertices $\gamma_{A_{1g}}, \gamma_{B_{1g}}$ and the parameters b_0, b_1. We assume that b_0 and b_1 are small: $\Delta_0 b_0, \Delta_0 b_1 \ll 1$; therefore A_{1g} has neglegible dependence on b_0, b_1. Since the $\mathcal{I}_n(x)$ are only functions of frequency $x = \omega/2\Delta_0 + i\eta$, the susceptibilities $\chi_{A_{1g}A_{1g}}, \chi_{B_{1g}B_{1g}}$ are functions of: $\chi_{A_{1g}A_{1g}}(\gamma_0, \gamma_1, \gamma_2, \eta, \Delta_0, \omega)$, $\chi_{B_{1g}B_{1g}}(\gamma_b, \eta, \Delta_0, \omega)$.

As a first step of fitting, we extract the parameters $(\gamma_b, \eta, \Delta_0)$ from the B_{1g} signal (see Fig. 4a). We use a Trust Region Reflective (trf) algorithm (scipy.optimize. least_squares) to fit the data. Electronic lifetime and the superconducting order parameter are characterized by η and Δ_0, which are expected to be shared amongst all response functions.

With the knowledge of η and Δ_0 we proceed to fit the A_{1g} pair-breaking response to acquire (γ_1, γ_2) (see Fig. 4b). No information of γ_0 can be obtained due to the complete screening of the isotropic A_{1g} component.[9]

We have known parameters $(\eta, \Delta_0, \gamma_1, \gamma_2)$ and free parameters (b_0, b_1, γ_0) in fitting the Higgs response (see Fig. 4c). These unknown values control the magnitude and lineshape of the Higgs Raman response function. It is well known that the diamagnetic response of the Higgs mode is small due to the approximate particle-hole symmetry near the Fermi surface. In a non-equilibrium experiment, however, redistribution of particles in response to an external optical pump will influence the state occupation, and thus the Raman signal magnitude. After 3 ps, the electronic degrees of freedom have relaxed to a quasi-equilibrium state, as can be seen by the establishment of thermal distribution on both Stokes and anti-Stokes sides except for the Higgs response. We therefore assume that the susceptibilities in the pumped states are well approximated by their equilibrium counterparts. In our equilibrium-calculation carried out in the clean limit we find that the Higgs susceptibility is about 3 orders of magnitudes weaker compared to the PB peak. This is due to the weak breaking of particle-hole symmetry in our model, which assumes a weak linear deviation in the Raman vertex and the energy dependence of the density of states.

S.3 Population Inversion

The Higgs modes are the lowest-energy collective excitations of the superconductor in the Cooper channel. In addition, the Higgs modes are metastable. This leads to a three-level picture of population inversion as

schematically shown in Fig. S9, motivating the NEARS experiment and outlining a basic understanding of the NEARS mechanism.

The rate equation for a three-level system [15] is given as

$$\frac{(N_2 - N_1)}{N} = \frac{(1 - \frac{\tau_{32}}{\tau_{21}}) \cdot W_p \tau_{21} - 1}{(1 - \frac{\tau_{32}}{\tau_{21}}) \cdot W_p \tau_{21} + 1} \tag{S23}$$

with N representing the total number of particles, N_1 being the number of particles in the ground state, and N_2 the measure for population of the metastable energy state (here the Higgs state). Via pumping, state 3 will be populated (Quench of the Mexican hat), with a short lifetime τ_{32}, subsequently populating the state 2 with a longer lifetime $\tau_{21} > \tau_{32}$. Assuming τ_{32} is much smaller than τ_{21} leads to $\frac{\tau_{32}}{\tau_{21}} \to 0$. W_p represents a measure of the strength of the pump, or $W_p \tau_{21} \propto F$ with F as pump fluence.

Population inversion occurs if $N_2 > N_1$, and $\frac{(N_2 - N_1)}{N} > 0$. Therefore, the anti-Stokes intensity of the metastable Higgs excitation, which corresponds to the anti-Stokes NEARS difference intensity shown in Fig. 2 of the main text, scales with the population ratio following

$$I_{AS} \begin{cases} \propto \frac{(N_1 - N_2)}{N} = \frac{F/F_{\text{crit}} - 1}{F/F_{\text{crit}} + 1} & \text{for } F > F_{\text{crit}} \to N_2 > N_1 \\ = 0 & \text{for } F < F_{\text{crit}} \to N_2 < N_1 \ . \end{cases} \tag{S24}$$

In fact, the integrated NEARS difference intensities on the anti-Stokes side (see Fig. 2c, d, and e) as a function of fluence are in agreement with eq. S24 as shown in Fig. S9b and Fig. 2e. F_{crit} is the critical fluence at which population inversion is initiated. Fitting eq. S24 to the experimental NEARS intensity integrals (see Fig. 2e main text and Fig. S9) results in F_{crit} of 21.7 ± 5.3 µJ cm^{-2} for A_{1g} and 31.6 ± 2.3 µJ cm^{-2} for B_{1g} symmetry.

S.4 Higgs Response in Ginzburg-Landau Theory

We describe the charged bosonic condensate using a Klein-Gordon like Lagrangian.[16, 1] The potential can be calculated with a Ginzburg-Landau Mexican-hat potential $F(\Psi) = \alpha|\Psi|^2 + \frac{\beta}{2}|\Psi|^4$ ($\alpha < 0$). For small fluctuations of the Higgs amplitude (H) around the ground state $|\Psi_0| = \sqrt{\frac{-\alpha}{\beta}}$ the Lagrangian can be written as [13]

$$\mathcal{L}^{KG} = (\partial_\mu H)(\partial^\mu H) + 2\alpha H^2 - \frac{1}{4} F_{\mu\nu} F^{\mu\nu} + q^2 \Psi_0^2 A_\mu A^\mu + 2q^2 \Psi_0 A_\mu A^\mu H \ , \tag{S25}$$

with the Cooper-pair charge $q = -2e$ and the electromagnetic field tensor $F_{\mu\nu} = \partial_\mu A_\nu - \partial_\nu A_\mu$. Due to the Anderson-Higgs mechanism, phase fluctuations (Θ) are no longer present in this expression.[17, 18] Instead, the low energy excitation spectrum in the condensate is only characterized by the Higgs mode. The photon field acquires a mass term leading to the Meißner effect when evaluating the Euler-Lagrange equations for the vector potential.[16] Furthermore, there is no linear coupling of the Higgs amplitude to

a vector potential. The Higgs mode is Raman active and couples quadratically to the vector potential. We can calculate the equation of motion for the Higgs mode by using the Euler-Lagrange equations and we find in the *optical* $q \to 0$ limit

$$\left(\frac{d^2}{dt^2} + 2|\alpha|\right) H(t) = e^2|\Psi_0|A^2, \tag{S26}$$

where we have set the electrostatic potential to 0.

In order to account for the non-equilibrium experimental conditions, we can quench the order parameter within the Ginzburg-Landau theory by quenching α, β, or both of them, since Ψ_0 itself depends on the ratio of $\sqrt{\frac{|\alpha|}{\beta}}$. If we quench α we will change the frequency of the Higgs mode to lower energy and reduce the coherence length $\xi = \sqrt{\hbar^2/(|\alpha|4m^*)}$ of the Cooper pairs, which is not observed in the NEARS experiment as shown in Fig. 2f. If we quench β, we will reduce the superfluid density and not change the frequency of the Higgs mode. This case fits our experimental observations and has a particularly simple solution. We can calculate the Green's function in the $\mathbf{q} \to 0$ case by assuming a δ function-like quench due to a change in β. We then obtain the following Green's function:

$$G_H(\omega) = \frac{1}{\omega^2 - 2|\alpha| + i\gamma\omega} = \frac{-i\gamma\omega}{(\omega^2 - 2|\alpha|)^2 + (\gamma\omega)^2} + \frac{(2|\alpha| - \omega^2)}{(\omega^2 - 2|\alpha|)^2 + (\gamma\omega)^2}\ , \tag{S27}$$

where we have added a phenomenological damping γ. This is identical to the Green's function of a harmonic oscillator with an eigenfrequency $\omega_0^2 = 2|\alpha|$.

It has been shown that in non-equilibrium the response function determining the Raman intensity $I(\omega)$ is proportional to the spectral function, i.e. the imaginary part of the Green's function (eq. S27).[19, 20] Accordingly, in the proximity of the Higgs-mode energy we approximate the Raman intensity as

$$I(\omega) = I_0 \frac{\gamma\omega}{(\omega^2 - 2|\alpha|)^2 + (\gamma\omega)^2}, \tag{S28}$$

where I_0 summarizes proportionalities to statistical factors, the strength of the quench, the excited state fraction of the experiment.

This is effectively a Lorentzian line shape with a strength of the response being proportional to the strength of the quench. In Ginzburg-Landau theory there is a direct connection between the energy of the Higgs mode encoded in α and the coherence length of a superconductor $\xi = \sqrt{\hbar^2/(|\alpha|4m^*)}$ as well as the penetration depth $\lambda = \sqrt{m^*c^2/(4\pi e^2\Psi_0^2)}$.[21] A quench of Ψ by β changes the penetration depth but leaves the coherence length constant, whereas the other two scenarios change both coherence length and penetration depth. In a strong coupling superconductor with a short coherence length we can expect our results to be close to a β quench, i.e. changing the suprafluid density alone. In more traditional weak coupling superconductors we would expect a change in position of the Higgs mode as α gets quenched. In mean field weak coupling approximation α is tied to 2Δ.

We fit the experimentally derived Higgs mode (see Fig. 2 main text) using eq. S28. The result is presented in Fig. 2c and d. In order to achieve consistent units in our experimental fit, we apply ω, γ, and α in units

of $2\Delta_0$, as determined by fitting the B_{1g} pair-breaking peak (see S.2.3, and Fig. 4 a). With this, the fit of the A_{1g} Higgs data at a fluence of 113 µJ cm^{-2} results in $2|\alpha| = (0.165 \pm 0.004)\ 2\Delta_0 = 10.07 \pm 0.24$ meV. This corresponds to an energy of the Higgs mode of $\omega_H = \sqrt{2|\alpha|} = 0.41 \cdot 2\Delta_0 = 25$ meV. Using established values m*/me for optimally-doped cuprates of the order of $m^* = 10\ m_e$,[22] we obtain in-plane coherence lengths of smaller than 5 nm.

S.5 Equilibrium Temperature Calculation

Raman spectroscopy enables the determination of an effective equilibrium sample temperature by the linking of Stokes and anti-Stokes spectra via the temperature-dependent Bose-function and thus makes a subtraction of laser-heating contributions from the original data possible. The effective temperature was identified using an algorithm based on the following equation:[23]

$$\frac{I_{\mathrm{AS}}(\Delta E)}{I_{\mathrm{S}}(\Delta E)} = \left(\frac{E_l + \Delta E}{E_l - \Delta E}\right)^4 \exp\left(-\frac{\Delta E}{k_{\mathrm{B}}T}\right) \tag{S29}$$

I_{AS} and I_{S} describe the measured anti-Stokes and Stokes Raman intensities as functions of Raman shift, E_l refers to the photon energy of the incident probe laser (3.09 eV in this study), ΔE is the Raman shift, k_{B} describes the Boltzmann constant, and T is the effective equilibrium temperature. Fig. S8 (a) shows the measured Stokes data (blue) and anti-Stokes data (red) together with the Stokes data mirrored to the anti-Stokes side by equation S29 (black). The base temperature for this measurement was 293 K (room temperature), and an effective temperature of 302 K had to be considered to achieve agreement between Stokes and anti-Stokes spectra. This corresponds to a heating of 9 ± 0.25 K for a probe laser power of 4.8 mW. Fig. S8 (b) shows the difference of Stokes and anti-Stokes integral as a function of heating for the exemplary measurement shown in (a) and defines the best estimate of the effective sample temperature as the minimum of the difference. To do this, we evaluate the integral difference in the energy region around two dominant phonons, as marked in Fig. S8 (a). To value the higher-energy integral with respect to the drastic reduction in anti-Stokes intensity as a function of energy due to the Bose function $n(\omega, T)$, we weight the higher-energy integral with the ratio $n(\omega = 14.4\,\mathrm{meV}, T)/n(\omega = 57.5\,\mathrm{meV}, T)$ (see Fig. S8). For the heating caused by the pump, the fluence dependent data set was used to define a heating rate (K/mW). As shown in Fig. S8 (c), we find a heating rate of 3 ± 0.5 K/mW for the pump. This leads to errors between 2.5 K and 13.75 K for the applied pump fluences (5 to 27 mW). The highest pump fluence at 113 µJ cm^{-2} (27 mW), therefore, corresponds to an effective equilibrium temperature of 98 ± 13.75 K, which equals T_C within its error bar. We validate this result in the superconducting state by analyzing the strength of the superconductivity-induced features of the Stokes spectra as a function of fluence. Fig. S12 shows the ratios of Stokes Raman spectra at 8 K base temperature divided by the 100 K probe-only data. As a function of fluence, the pair-breaking feature gets suppressed and gap-filling occurs. To obtain a measure for the system's state, we evaluate the integral of the absolute value of these ratios compared to the normal state, in

.

Table S2: Laser power measured by PowerMax USB PS19 (Coherent, see power meter in Fig. S1), spot size at sample position measured by a DFK 23GM021 industrial camera (The Imaging Source) with a pixel size of 3.75 µm, repetition rate of the used Tsunami system with a pulse width of FWHM = 1.2 ± 0.1 ps, corresponding fluence for probe and pump, and effective equilibrium temperature following Fig. S8

	**Laser Power**	**Spot Area**	**Rep. Rate**	**Fluence**	**Effective Temp.**
**Probe**	4.75 ± 0.15 mW	169.23 ± 10.88 μm^2	80 MHz	35.08 ± 1.15 µJ cm^{-2}	17 ± 0.25 K
**Pump**	5.0 ± 0.1 mW	298.65 ± 19.19 μm^2	80 MHz	20.97 ± 0.93 µJ cm^{-2}	32 ± 2.75 K
	10.0 ± 0.1 mW	298.65 ± 19.19 μm^2	80 MHz	41.95 ± 2.27 µJ cm^{-2}	47 ± 5.25 K
	14.0 ± 0.1 mW	298.65 ± 19.19 μm^2	80 MHz	58.72 ± 3.35 µJ cm^{-2}	59 ± 7.25 K
	18.0 ± 0.1 mW	298.65 ± 19.19 μm^2	80 MHz	75.50 ± 4.43 µJ cm^{-2}	71 ± 9.25 K
	27.0 ± 0.1 mW	298.65 ± 19.19 μm^2	80 MHz	113.25 ± 6.86 µJ cm^{-2}	98 ± 13.75 K

other words $\int |8\text{K data} \,/\, 100\text{ K probe data} - 1|$. This is shown in the inset of Fig. S12. The 100 K baseline intersects with a linear regression fit at a fluence of 139.3 ± 28.5 µJ cm^{-2}.

In summary, from both methods we conclude that (I) the sample pumped with a fluence of 113 µJ cm^{-2} has an effective equilibrium temperature of 98 ± 13.75 K (see Table S2), and (II) T_C is reached at a fluence of 139.3 ± 28.5 µJ cm^{-2}. Both error bars overlap. Since we observe a clear pair-breaking feature at our highest pump fluence (see Fig. S12 and Fig. S6), we conclude that the sample remains in its superconducting state during our measurements.

S.6 Data Parameterization

Stokes Raman intensities are described by the following fit function:

$$I_\mathrm{S} = (n(\omega,T)+1)\cdot\left[y_0\cdot\tanh\left(\frac{\omega}{\omega_\mathrm{c}}\right) + \frac{A_{2\Delta}\,\omega\,\tilde{\Gamma}_{2\Delta}}{(\omega^2-\omega_{0,2\Delta}^2)^2+\tilde{\Gamma}_{2\Delta}^2\omega^2} + \sum_m \frac{A_m\,\omega\,\tilde{\Gamma}_m}{(\omega^2-\omega_{0,m}^2)^2+\tilde{\Gamma}_m^2\omega^2}\right] \quad \text{(S30)}$$

where $(n(\omega,T)+1)$ represents the thermal population factor (Bose-function, see Fig. S4):

$$n(\omega,T)+1 = \frac{1}{\exp\left(\frac{\hbar\omega}{k_\mathrm{B}T}\right)-1}+1 = \frac{1}{1-\exp\left(-\frac{\hbar\omega}{k_\mathrm{B}T}\right)} \quad \text{(S31)}$$

In the superconducting state, the gap feature is parameterized by a tanh-function $y_0\cdot\tanh\left(\frac{\omega}{\omega_\mathrm{c}}\right)$ with the critical frequency ω_c.[11] According to [11], the pair-breaking peak is described by a Lorentzian, with $A_{2\Delta}$ being the amplitude, $\tilde{\Gamma}_{2\Delta}$ the damping and $\omega_{0,2\Delta}$ corresponding to the gap energy. This parameterization of the pair-breaking feature (tanh + Lorentzian) represents a model-independent analysis of the electronic susceptibility. The quality of the electronic features derived from our fit model and parameterized by a tanh-function together with Lorentzian can be evaluated by comparison with a microscopic theory as shown in Fig. 4 (main text). The electronic responses are extracted from the data by subtracting the phonons as determined by the fit (eq. S30, see also Fig. S10). In fact, the parameterization results show perfect agreement with the respective theory calculations. The phonons ($m = 9$) are fitted as Lorentzians, where A_m denotes the amplitude, $\tilde{\Gamma}_m$ is the phonon damping, and $\omega_{0,m}$ is the phonon frequency of the mth phonon.

To parameterize the anti-Stokes spectra, phonon frequencies and widths, background, and pair-breaking feature are kept constant between Stokes and anti-Stokes fits. The Bose-function changes from $(n(\omega,T)+1)$

to $n(\omega, T)$ (see Fig. S4). Phonon amplitudes are allowed to adapt to the measured anti-Stokes phonon intensities.

S.7 Higgs Mode and Other SC Excitations

We now discuss our results in the context of the Higgs mode and how it can be discriminated from alternative excitations of the superconductor. The important candidates are the pair-breaking (quasiparticle) excitations, the Josephson plasmon modes, and the Bardasis-Schrieffer modes. *Pair-breaking excitation:* This is the dominant feature on the Stokes side of the spectra. It occurs due to the breaking of Cooper-pairs and generates quasiparticles in the single-particle channel, i.e. holes in Bi-2212 (see Fig. 1c). It is symmetry-dependent due to the inherent Coulomb screening, which is fully symmetric and affects only the A_{1g} channel, reducing the A_{1g} susceptibility and shifting the excitation to lower energies compared to B_{1g} symmetry (see Fig. 4). In S.2 and the main text (Fig. 4), we discuss in detail the pair-breaking response which is measured by NEARS and calculated within the framework of a microscopic theory. *Excitations of in-plane Josephson plasmon modes:* They exhibit A_{1g} and B_{1g} symmetry.[24] However, we would expect the Josephson plasmon to exhibit an excitation energy that shifts to zero frequency as we increase the pump fluence.[24] Even our strongly-pumped A_{1g} data do not show this behavior. Therefore, they can be ruled out as well. *Bardasis-Schrieffer mode:* This mode represents a subdominant pairing channel that could be activated by pumping the SC state. Its excitation energy would be below the binding energy 2Δ of the dominant pairing channel and this mode would also not be subject to screening. Hence, these two arguments do not rule out this mode. The Bardasis-Schrieffer mode would leave a distinct signature on the Stokes side, as observed in pnictides.[25, 26, 27] However, our Stokes data shows no evidence of this mode. Moreover, our analysis, which relies on comparison of Stokes and anti-Stokes intensity, inherently accounts for its potential presence. Furthermore, to the best of our knowledge, there is no evidence of Bardasis-Schrieffer modes in cuprates. In a pump-probe experiment, a pump-activated Bardasis-Schrieffer mode would typically shift to lower energies with increasing pump fluence.[28] However, we observe that the energy of the NEARS feature remains nearly constant across different fluences. In addition, NEARS is driven by population inversion, which amplifies the anti-Stokes signal relative to the Stokes signal (see Fig. S9). For this to occur, the observed mode must be metastable over several picoseconds. This serves as a strong argument for the Higgs mode's relevance in explaining our observations, as its metastable nature facilitates population inversion. [1] Additionally, all our data and in particular of the SC gap feature confirm that we remain in the superconducting state. This leaves the Higgs mode as the best explanation for the NEARS feature.

S.8 Raman Selection Rules

Disorder and orthorhombic distortions play a significant role in shaping the Raman spectra and selection rules of Bi-2212.[29, 30, 31] Disorder, particularly in the buffer layers, can induce additional Raman-active

[1] In the presence of nearly degenerate pairing channels, a metastable Bardasis-Schrieffer mode may, in principle, arise.[28] Nonetheless, to the best of our knowledge, and despite more than three decades of research on high-T_C superconductors, there is still no convincing evidence supporting such a scenario in cuprates.

phonon modes, modifying spectral features and enhancing anisotropic charge fluctuations. This disorder-induced phonon behavior is further influenced by doping, as phonon modes in Bi-2212 shift due to variations in carrier concentration, leading to spectral weight enhancement in specific symmetries.[29] Orthorhombic distortions break the ideal tetragonal symmetry and modify Raman selection rules by lifting degeneracies and enabling new phonon activations as also seen by resonance Raman studies.[11] Most importantly, resonance Raman studies demonstrate the appearance of disorder-induced phonon modes in the UV spectral range close to the charge-transfer transition and in general the strong dependence of the Raman matrix element on incident photon energy, leading to an amplification of the 2Δ excitation.[11] Fig. S10 b) shows the Raman spectra for all three measured symmetry configurations. We observe a clear phonon suppression when comparing $A_{2g}+B_{1g}$ and $A_{2g}+B_{2g}$ with $A_{1g}+B_{2g}$ data demonstrating that several phonons have A_{1g} character. This symmetry-dependent behavior of phonons in Bi-2212 in the UV spectral range is in line with previous observations.[11] Most importantly, our NEARS measurements confirm the expected selection rules for the Higgs modes. In this experimental configuration, the Higgs mode is predicted to be excited in the A_{1g} and B_{1g} symmetries but not in the $A_{2g} + B_{2g}$ configuration [32], as demonstrated in Fig. S11.

References

[1] Pekker, D. & Varma, C. M. Amplitude/Higgs modes in condensed matter physics. *Annual Review of Condensed Matter Physics* **6**, 269 – 297 (2015). URL https://doi.org/10.1146/annurev-conmatphys-031214-014350.

[2] Nambu, Y. Quasi-particles and gauge invariance in the theory of superconductivity. *Phys. Rev.* **117**, 648 – 663 (1960). URL https://doi.org/10.1103/PhysRev.117.648.

[3] Peskin, M. & Schroeder, D. *An Introduction To Quantum Field Theory* (Taylor and Fracis Group, 1995).

[4] Zirnbauer, M. R. Particle–hole symmetries in condensed matter. *Journal of Mathematical Physics* **62** (2021). URL https://doi.org/10.1063/5.0035358.

[5] Chiu, C.-K., Teo, J. C. Y., Schnyder, A. P. & Ryu, S. Classification of topological quantum matter with symmetries. *Rev. Mod. Phys.* **88**, 035005 (2016). URL https://doi.org/10.1103/RevModPhys.88.035005.

[6] Markiewicz, R. S., Sahrakorpi, S., Lindroos, M., Lin, H. & Bansil, A. One-band tight-binding model parametrization of the high-T_C cuprates including the effect of k_z dispersion. *Phys. Rev. B* **72**, 054519 (2005). URL https://doi.org/10.1103/PhysRevB.72.054519.

[7] Devereaux, T. P. & Hackl, R. Inelastic light scattering from correlated electrons. *Rev. Mod. Phys.* **79**, 175 – 233 (2007). URL https://doi.org/10.1103/RevModPhys.79.175.

[8] Hwang, J. Superconducting coherence length of hole-doped cuprates obtained from electron–boson spectral density function. *Sci Rep* **11**, 11668 (2021). URL https://doi.org/10.1038/s41598-021-91163-w.

[9] Devereaux, T. P. & Einzel, D. Electronic Raman scattering in superconductors as a probe of anisotropic

electron pairing. *Phys. Rev. B* **51**, 16336 – 16357 (1995). URL https://doi.org/10.1103/PhysRevB.51.16336.

[10] Monien, H. & Zawadowski, A. Theory of Raman scattering with final-state interaction in high-T_C BCS superconductors: Collective modes. *Phys. Rev. B* **41**, 8798–8810 (1990). URL https://doi.org/10.1103/PhysRevB.41.8798.

[11] Budelmann, D. *et al.* Gaplike excitations in the superconducting state of $Bi_2Sr_2CaCu_2O_8$ studied by resonant Raman scattering. *Phys. Rev. Lett.* **95**, 057003 (2005). URL https://doi.org/10.1103/PhysRevLett.95.057003.

[12] Cea, T. & Benfatto, L. Nature and Raman signatures of the Higgs amplitude mode in the coexisting superconducting and charge-density-wave state. *Phys. Rev. B* **90**, 224515 (2014). URL https://doi.org/10.1103/PhysRevB.90.224515.

[13] Puviani, M., Schwarz, L., Zhang, X.-X., Kaiser, S. & Manske, D. Current-assisted Raman activation of the Higgs mode in superconductors. *Phys. Rev. B* **101**, 220507 (2020). URL https://doi.org/10.1103/PhysRevB.101.220507.

[14] Cea, T., Castellani, C. & Benfatto, L. Nonlinear optical effects and third-harmonic generation in superconductors: Cooper pairs versus Higgs mode contribution. *Phys. Rev. B* **93**, 180507 (2016). URL https://link.aps.org/doi/10.1103/PhysRevB.93.180507.

[15] Shimoda, K. *Introduction to Laser Physics*. Springer Series in Optical Sciences (Springer Berlin Heidelberg, 2013). URL https://books.google.de/books?id=yqPxCAAAQBAJ.

[16] Greiter, M. Is electromagnetic gauge invariance spontaneously violated in superconductors? *Annals of Physics* **319**, 217–249 (2005). URL https://doi.org/10.1016/j.aop.2005.03.008.

[17] Anderson, P. Coherent excited states in the theory of superconductivity: gauge invariance and the Meissner effect. *Phys. Rev.* **110**, 827 – 835 (1958). URL https://doi.org/10.1103/PhysRev.110.827.

[18] Anderson, P. W. Plasmons, gauge invariance, and mass. *Phys. Rev.* **130**, 439–442 (1963). URL https://doi.org/10.1103/PhysRev.130.439.

[19] Freericks, J. & Kemper, A. F. What do the two times in two-time correlation functions mean for interpreting tr-ARPES? *Journal of Electron Spectroscopy and Related Phenomena* **251**, 147104 (2021). URL https://doi.org/10.1016/j.elspec.2021.147104.

[20] Wang, Y., Devereaux, T. P. & Chen, C.-C. Theory of time-resolved Raman scattering in correlated systems: Ultrafast engineering of spin dynamics and detection of thermalization. *Phys. Rev. B* **98**, 245106 (2018). URL https://link.aps.org/doi/10.1103/PhysRevB.98.245106.

[21] Cyrot, M. Ginzburg-landau theory for superconductors. *Reports on Progress in Physics* **36**, 103 (1973). URL https://dx.doi.org/10.1088/0034-4885/36/2/001.

[22] Legros, A. *et al.* Universal T-linear resistivity and Planckian dissipation in overdoped cuprates. *Nature Phys.* **15**, 142–147 (2019). URL https://doi.org/10.1038/s41567-018-0334-2.

[23] Bock, A. Laser heating of $YBa_2Cu_3O_7$ films in Raman experiments. *Phys. Rev. B* **51**, 15506 – 15518

(1995). URL https://doi.org/10.1103/PhysRevB.51.15506.

[24] Gabriele, F., Udina, M. & Benfatto, L. Non-linear Terahertz driving of plasma waves in layered cuprates. *Nat Commun* **12**, 752 (2021). URL https://doi.org/10.1038/s41467-021-21041-6.

[25] Scalapino, D. J. & Devereaux, T. P. Collective d-wave exciton modes in the calculated Raman spectrum of Fe-based superconductors. *Phys. Rev. B* **80**, 140512 (2009). URL https://doi.org/10.1103/PhysRevB.80.140512.

[26] Bardasis, A. & Schrieffer, J. R. Excitons and plasmons in superconductors. *Phys. Rev.* **121**, 1050 – 1062 (1961). URL https://doi.org/10.1103/PhysRev.121.1050.

[27] Chubukov, A. V., Eremin, I. & Korshunov, M. M. Theory of Raman response of a superconductor with extended s-wave symmetry: Application to the iron pnictides. *Phys. Rev. B* **79**, 220501 (2009). URL https://doi.org/10.1103/PhysRevB.79.220501.

[28] Sun, Z., Fogler, M. M., Basov, D. N. & Millis, A. J. Collective modes and terahertz near-field response of superconductors. *Phys. Rev. Res.* **2**, 023413 (2020). URL https://doi.org/10.1103/PhysRevResearch.2.023413.

[29] Munnikes, N. *et al.* Pair breaking versus symmetry breaking: Origin of the Raman modes in superconducting cuprates. *Phys. Rev. B* **84**, 144523 (2011). URL https://doi.org/10.1103/PhysRevB.84.144523.

[30] Hackl, R. *et al.* Raman study of ordering phenomena in copper–oxygen systems. *Journal of Physics and Chemistry of Solids* **67**, 289–293 (2006). URL https://www.sciencedirect.com/science/article/pii/S0022369705005159. Spectroscopies in Novel Superconductors 2004.

[31] Boulesteix, C., Hewitt, K. C. & Irwin, J. C. Temperature-induced frequency shift of the raman-active cuo2 planar oxygen vibrational modes of bi-2212 related to a change of the cu-o bonding. *Journal of Physics: Condensed Matter* **12**, 9637 (2000). URL https://dx.doi.org/10.1088/0953-8984/12/46/311.

[32] Schwarz, L. *et al.* Classification and characterization of nonequilibrium Higgs modes in unconventional superconductors. *Nat Commun* **11**, 287 (2020). URL https://doi.org/10.1038/s41467-019-13763-5.

[33] Schulz, B. *et al.* Fully reflective deep ultraviolet to near infrared spectrometer and entrance optics for resonance Raman spectroscopy. *Rev. Sci. Instrum.* **76**, 073107 (2005). URL https://doi.org/10.1063/1.1946985.

[34] Perfetti, L. *et al.* Ultrafast electron relaxation in superconducting $Bi_2Sr_2CaCu_2O_{8+\delta}$ by time-resolved photoelectron spectroscopy. *Phys. Rev. Lett.* **99**, 197001 (2007). URL https://doi.org/10.1103/PhysRevLett.99.197001.

Supplementary Figures

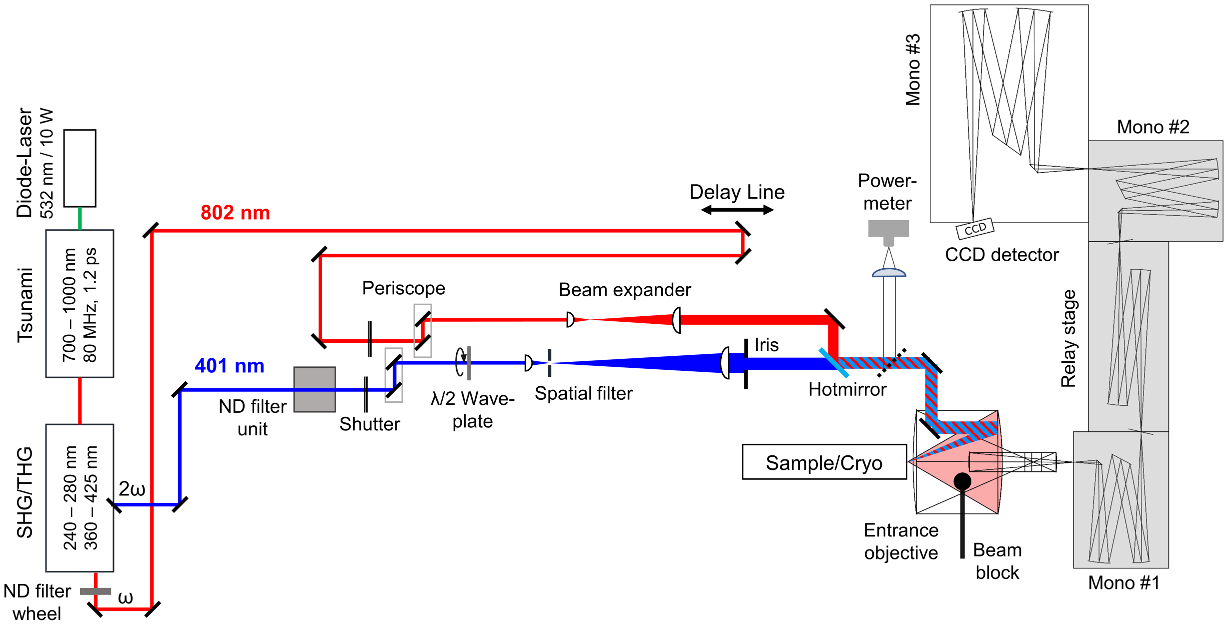

Fig. S1: Schematic view on the Raman setup. A Tsunami Ti:Sapphir system with a pulse duration of 1.2 ps and a repetition rate of 80 MHz is used as laser source at a fundamental wavelength of 802 nm (pump). From a second harmonic generation (SHG) unit, a 402 nm beam is used as the probe. The beam path includes ND filter units, shutters, a $\lambda/2$ waveplate to change the linear polarization of incidence light, a beam expander and spatial filter, a delay line for temporal overlap and scanning, and a hot mirror to overlay both beams. The entrance objective with its large numerical aperture of 0.5 is used to focus the light on the sample, and couples the Raman light into the first monochromator of the UT-3 spectrometer.[33] A beam block is used to block the specular reflex.

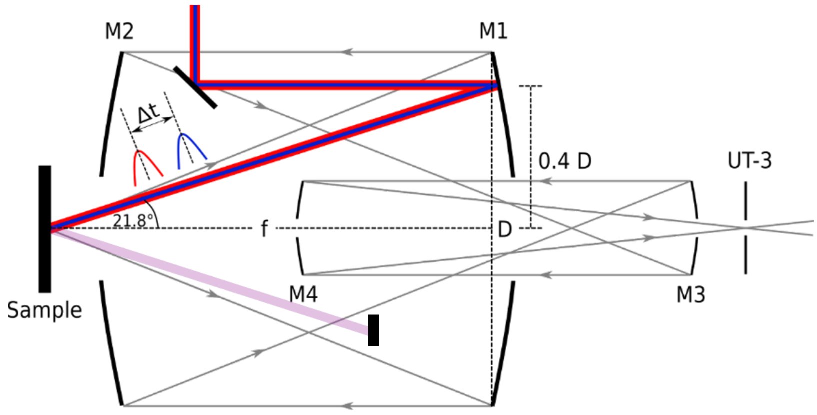

Fig. S2: Detailed view on the entrance objective of the UT-3. The Raman signal is collected with M1, collimated onto M2, which focuses the light in front of M3. This mirror collimates the light to provide a parallel beam section for the insertion of an analyzing beam cube. Pump and probe beam hit the sample with an angle of 21.8° to the vertical. By this, we apply a finite in-plane momentum to the sample, which causes symmetry breaking and activation of the Higgs mode in B_{1g} symmetry. A beam dump blocks the reflected light in 21.8°.

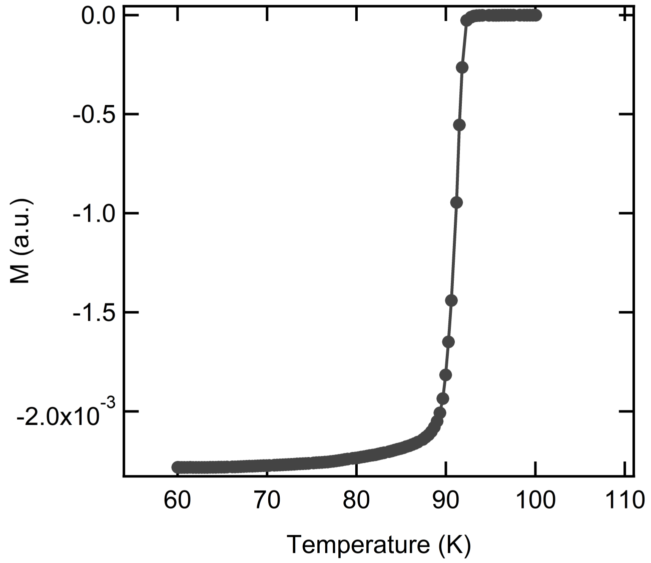

Fig. S3: Susceptibility curves of the measured Bi-2212 crystal with a T_c of 92 K.

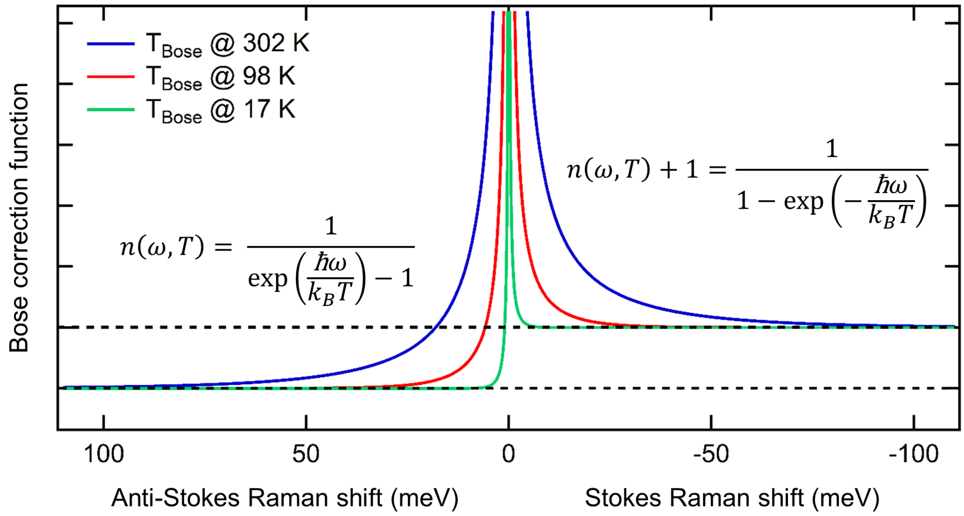

Fig. S4: Bose function for anti-Stokes Raman scattering ($n(\omega, T)$) und Stokes Raman scattering ($n(\omega, T)+1$) for three exemplary temperatures. On the Stokes side, the Bose function converges to 1 for high energies, resulting in a non-zero Raman intensity. In comparison, on the anti-Stokes side, the Raman intensity aspires to become zero. Depending on the thermal population, an experimental observation window at low Raman shifts exists.

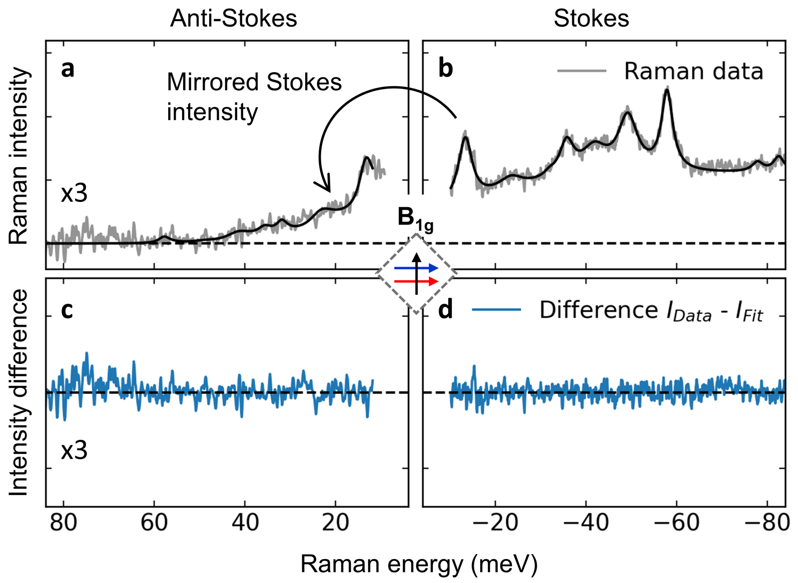

Fig. S5: Bi-2212 anti-Stokes (a) and Stokes (b) probe-only Raman intensity for B_{1g} geometry as a function of Raman shift at 100 K base temperature. The data is shown in gray. The black solid line represents a fit to the data, for which the Stokes Raman intensity (b) has been parameterized as described in section S.4. The Stokes intensity fit was then applied to the anti-Stokes side by using the anti-Stokes Bose function $n(\omega, T)$ instead of $n(\omega, T) + 1$. A temperature $T = 109$ K was used (100 K base temperature and 9 K probe heating, see Fig. S8). Phonon widths and frequencies were kept constant after fitting to the Stokes side. (c) and (d) show the difference between the data (gray) and the fit (black) shown in (a) and (b). The dashed line marks zero.

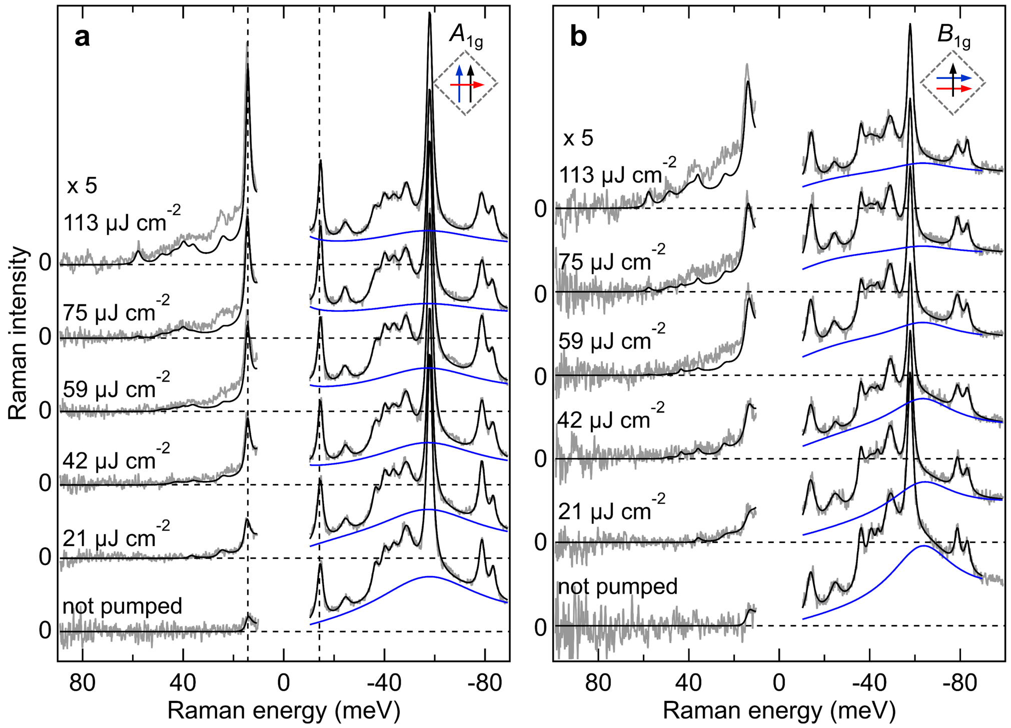

Fig. S6: Complete data sets corresponding to Fig. 2 of the main text. (a) A_{1g} Pump-probe Stokes and anti-Stokes Raman spectra at 8 K base temperature and a time delay of 3 ps for fluences between 0 µJ cm^{-2} (not pumped) and 113 µJ cm^{-2}. Anti-Stokes Raman intensities are multiplied with a factor of 5 for better visibility. Data is shown in gray and black solid lines represent fits to the Stokes Raman intensities. The dashed horizontal lines mark the zero intensity for the data sets at different fluences. The PB peak on the Stokes side is highlighted in blue. At fluences larger than 50 µJ cm^{-2} the soft quench overpopulates the Higgs excitation and additional spectral weight is detected on the anti-Stokes side. (b) Analogous data set for B_{1g} configuration.

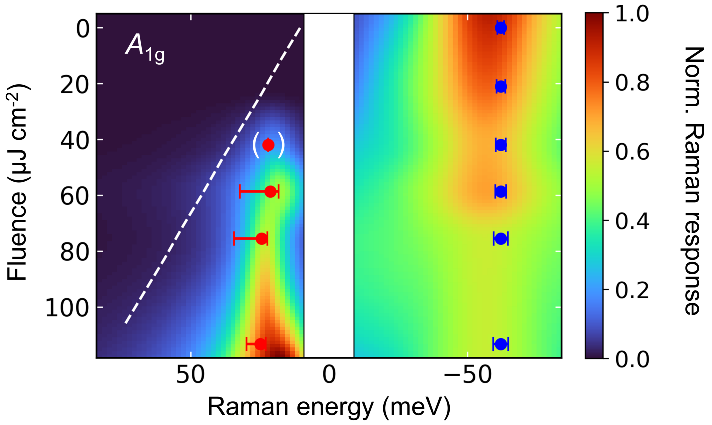

Fig. S7: Interpolated 2D color plot of the superconductivity-induced Raman susceptibilities obtained from the fit to the NEARS data in A_{1g} geometry. In the right (energy-loss) side, the pair-breaking feature from the Stokes data is shown (tanh-function plus Lorentzian as extracted from our fit to the Raman intensities). It decreases with increasing fluence. The blue data points represent the fit results (see Fig. 2 main text). Error bars are the standard uncertainty of the fitted frequency for the PB peak. On the left (energy-gain) side, one can observe the NEARS feature, representing the difference signal visible on the anti-Stokes side compared to the Stokes data. This difference signal can be identified, since Stokes and anti-Stokes data are linked via the Bose-function and, therefore, the Stokes data can be mirrored to the anti-Stokes side. The white dashed line represents the cutoff of the thermal observation window on the anti-Stokes side at the derived equilibrium temperatures. In correspondence with our instrumental resolution, we determine this cutoff to be at a critical energy as a function of effective sample temperature, where the Bose-function $n(\omega, T)$ brings the Raman intensity down to 10 %. The red data points represent the fit results of the NEARS feature frequency based on eq. S28. The error bars indicate the standard uncertainty of the fit, with asymmetry reflecting the increased signal-to-noise ratio on the higher-energy side due to the Bose function. With this, we derive the NEARS map (see Fig. 3 main text) as a superposition of the two features as a function of absolute value of the Raman energy, corresponding to the excitation energy.

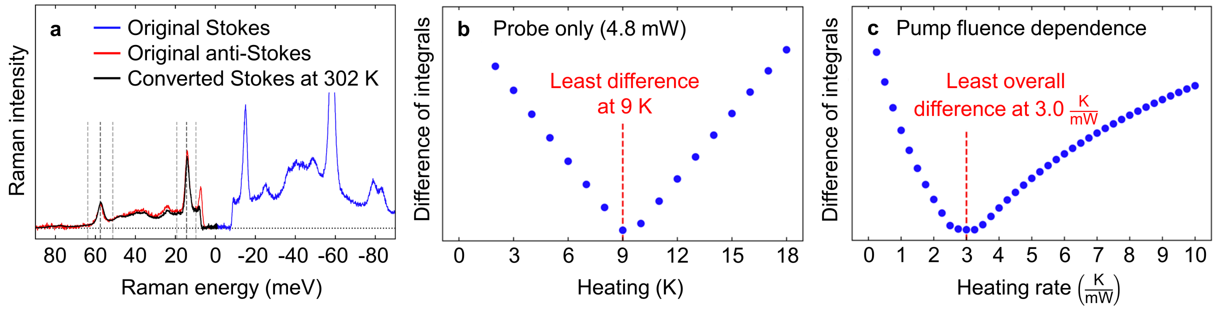

Fig. S8: (a) Converted probe-only Stokes spectrum (black) calculated from the original Stokes data (blue) according to eq. S29 at an effective temperature of 302 K (base temperature = 293 K). To determine the effective sample temperature of 302 K, the integral difference between the original anti-Stokes data (red) and the converted Stokes data around the dominant phonon modes for different temperatures was evaluated. Integral bounds are shown as vertical light gray dashed lines and the locations used for determining the thermal weighting factor for the high-energy integral are shown as gray dashed lines in the middle of the two integral bounds, respectively. Zero intensity is marked as horizontal dotted line. (b) Difference of the integrals of original anti-Stokes data and converted Stokes data as introduced in (a) as a function of assumed heating for the probe-only data. The effective heating is equal to the difference between the temperature used to convert the Stokes data to the anti-Stokes side and the base temperature of 293 K. One can clearly identify a minimum at a heating of 9 ± 0.25 K (302 K sample temperature). (c) Result of the algorithm performed on the entire series of fluence-dependent pump-probe measurements at a base temperature of 293 K, showing a heating rate of 3 ± 0.5 K/mW for the pump.

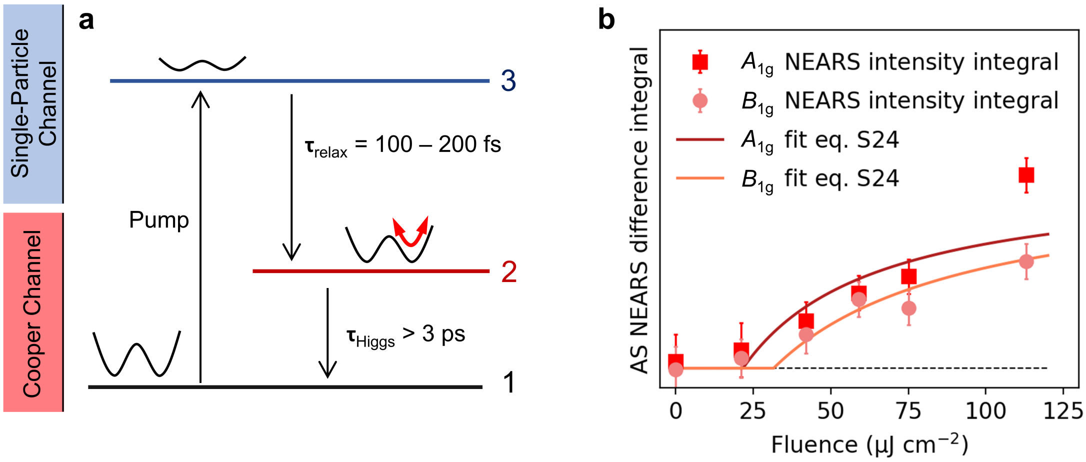

Fig. S9: (a) Cooper channel vs. single-particle channel in the NEARS experiment on Bi-2212 highlighting the specific excitation process activating the Higgs mode in the NEARS channel. The Mexican-Hat potential representing the superconducting ground state (1) is quenched upon pumping (3). Concomitantly, the reduction of the superfluid density and gap-filling occurs. On a time-scale of 100-200 fs[34] the Mexican-Hat potential relaxes. The holes from the single-particle channel form again bosonic Cooper pairs, which oscillate in the relaxing Mexican-Hat potential representing an excited and metastable Higgs state (2). This population inversion is probed by NEARS leading to a strongly enhanced Raman susceptibility on the anti-Stokes side. It is important to note that this represents a three-level system in which the excited transient Higgs state (N_2) has a higher population than the ground state (N_1) leading to population inversion and a stronger anti-Stokes than Stokes response. This is only possible in superconductors due to the interplay between single-particle and Cooper channels and the metastable character of the Higgs excitations as the lowest-energy collective excitation of the Cooper channel. Conventional excitations such as phonons cannot show this behavior. (b) Integrated anti-Stokes NEARS difference intensity (Higgs mode intensity) (see Fig. 2 main text) for A_{1g} (red squares) and B_{1g} (light red circles) symmetry, together with the strength of population inversion following equation S24 with a critical fluence of 21.7 ± 5.3 μJ cm^{-2} for A_{1g} (dark red solid line) and 31.6 ± 2.3 μJ cm^{-2} for B_{1g} symmetry (light red solid line). The dashed horizontal line marks zero. Error bars are determined based on the noise of the integrated data.

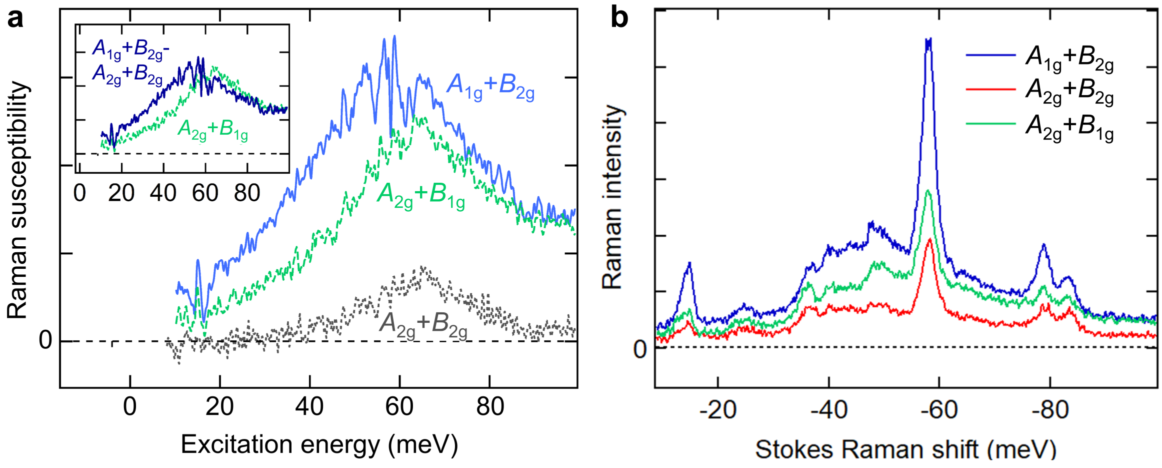

Fig. S10: (a) Raman susceptibilities of the pair-breaking feature in the experimental scattering configurations A_{1g}+B_{2g} (blue), A_{2g}+B_{1g} (green), and A_{2g}+B_{2g} (gray). To extract these curves from our Raman intensities, we corrected for the Bose function and subtracted all phonons as determined by our parameterization approach (see eq. S30). The inset shows the comparison between the electronic B_{1g} feature (green, A_{2g}+B_{1g}) together with the subtracted feature A_{1g}+B_{2g} - A_{2g}+B_{2g} (dark blue). (b) Stokes intensities (probe only) at 10 K for all three measured symmetry configurations. A clear suppression of phonon modes is observed in predominantly B_{1g} (green) and B_{2g} (red) symmetries compared to predominantly A_{1g} symmetry (blue). As demonstrated by Budelmann et al.[11], disorder-induced phonon modes emerge as a function of incident photon energy when probing charge-transfer excitation in Bi-2212.

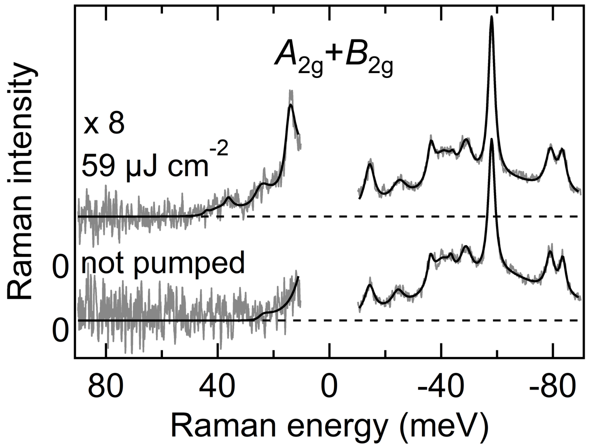

Fig. S11: A_{2g}+B_{2g} Stokes and anti-Stokes Raman data (gray) and fits as discussed in S.4 and Fig. 1 and 2 (main text). The bottom spectra show probe-only (equilibrium) data. For the upper spectra a pump fluence of 59 µJ cm^{-2} was applied. In contrast to the A_{1g} and B_{1g} data presented in the main text, we cannot identify a Higgs mode here. The anti-Stokes data in the puped state can be fully described by the phonons and electronic background fitted to the Stokes side. No difference occurs.

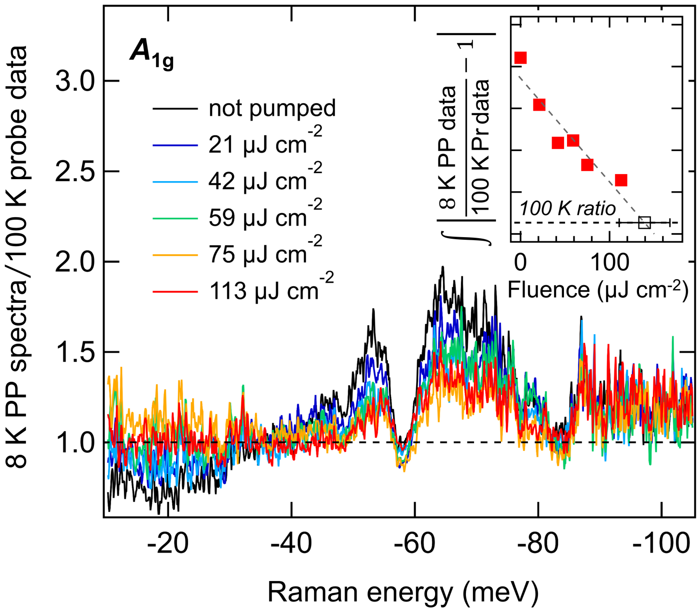

Fig. S12: Ratios between 8 K Stokes and 100 K Stokes Raman spectra. Bi-2212 A_{1g} Stokes Raman susceptibilities at 8 K base temperature and different pump fluences are divided by the 100 K non-pumped (probe-only) susceptibility. This comparison shows the pair-breaking feature around 60 meV and the superconducting gap below 30 meV (see also Fig. S13). As a function of pump fluence, the pair-breaking peak gets suppressed and gap-filling occurs. The dashed horizontal line marks the ratio of 1. The inset shows the integrated values for the absolute values of the displayed ratios ($\int$ |8 K PP spectra / 100 K Probe − 1|) representing a measure for the strength of the superconductivity-induced feature on the Stokes side. A linear regression fit intersects with the base line for the ratio determined in the normal state at a fluence of 139.3 ± 28.5 µJ cm^{-2} (black square with error bar in the inset). This is an alternative method to derive the effective sample temperature compared to T_C and it's result is in agreement with our method shown in Fig. S8. For the latter, we obtain an equilibrium temperature for the sample at 8 K base temperature and with a pump fluence of 113 µJ cm^{-2} of 98 ± 13.75 K, which corresponds to T_C within the error bar. Here, T_C is reached at a fluence of 139.3 ± 28.5 µJ cm^{-2}, which includes 113 µJ cm^{-2} within the error bar.

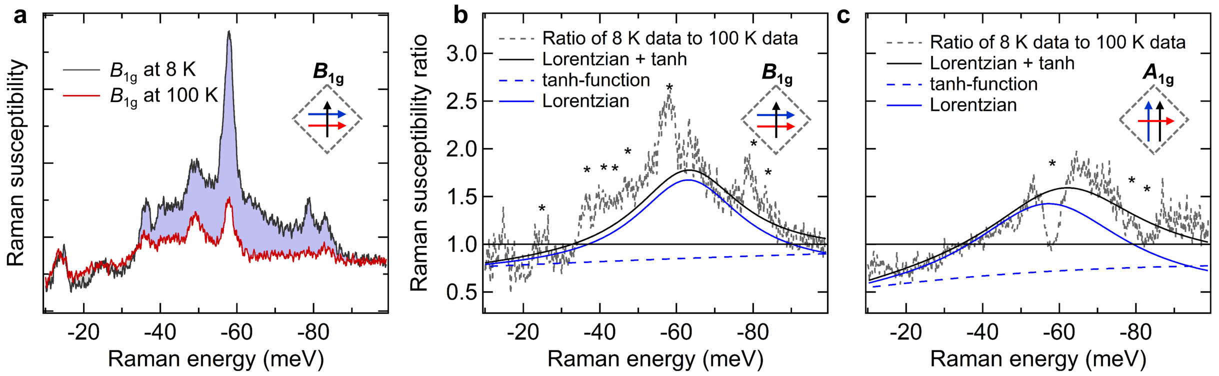

Fig. S13: (a) Bi-2212 B_{1g} Stokes Raman spectra (not pumped) at 8 K (gray) and 100 K (red) base temperatures. The comparison between 8 K and 100 K shows the pair-breaking feature (highlighted in blue) and a slight gap feature at this excitation wavelength of 400 nm (3.1 eV). This result is in agreement with previous studies.[11] (b) Ratio between 8 K and 100 K B_{1g} Raman susceptibilities as shown in (a) (gray dashed). The horizontal line marks the ratio of 1. Stars mark phonon positions which lead to artifacts in the ratio due to slight differences in phonon intensity and width at the different temperatures. A model-independent parameterization of the electronic susceptibility can be achieved via a tanh-function (dashed blue) together with a Lorentzian (solid blue). The sum of both curves is shown as black solid line. (c) Ratio of 8 K to 100 K data for A_{1g} symmetry. The parameterization of the electronic susceptibility is presented in the same way as shown in (b).

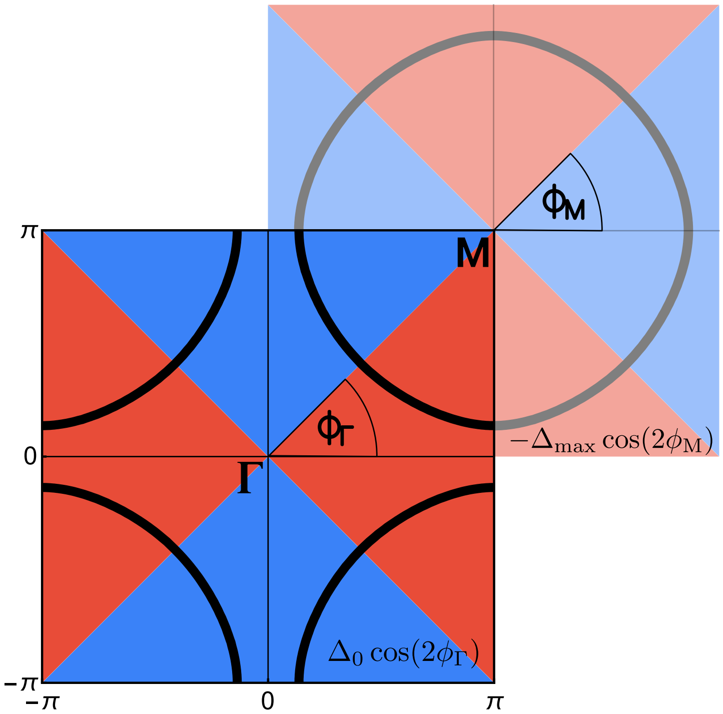

Fig. S14: Schematic view of the typical Fermi surface of a cuprate SC like Bi-2212. The d-wave symmetry of the gap gives an angular dependence in reciprocal space of the form $\Delta_\mathbf{k} = \Delta_0 \cos 2\phi$, the sign of which is indicated in red and blue. Due to the first Brillouin zone's periodicity, the Fermi surface can be viewed as connected around the point $M = (\pi, \pi)$. In terms of the angle defined around the M-point the gap is described by $\Delta_\mathbf{k} \approx -\Delta_{\max} \cos 2\phi$. The inversion of the absolute phase is irrelevant since it is gauge-dependent. The appearance of the new parameter $\Delta_{\max} \leq \Delta_0$ is due to the distance of the Fermi surface from the coordinate axes (when viewed around Γ), where $\cos 2\phi_\Gamma = 1$. However, for the M enclosing Fermi surface $\Delta_{\max}$ is the only physical gap amplitude and is therefore called Δ_0 as is conventional.
